# Meta-prediction of MTHFR gene polymorphism-mutations, air pollution, and risks of leukemia among world populations

**DOI:** 10.18632/oncotarget.13876

**Published:** 2016-12-10

**Authors:** Shin-Yu A. Lien, Lufei Young, Bih-Shya Gau, S. Pamela K. Shiao

**Affiliations:** ^1^ School of Nursing, College of Medicine, Chang Gung University, Taoyuan, Taiwan (R.O.C.); ^2^ Division of Endocrinology and Metabolism, Department of Internal Medicine, Chang Gung Memorial Hospital Linkou Branch, Taoyuan, Taiwan (R.O.C.); ^3^ College of Nursing, Augusta University, Augusta, Gerogia, USA; ^4^ School of Nursing, College of Medicine, National Taiwan University, Taipei, Taiwan (R.O.C.)

**Keywords:** meta-analysis, meta-prediction, methylenetetrahydrofolate reductase, leukemia, air pollution

## Abstract

The major objective of this study was to examine the association between *Methylenetetrahydrofolate Reductase* (*MTHFR*) polymorphisms and the risk of various types of leukemias across the lifespans of children and adults by using the meta-predictive techniques. The secondary objective was to examine the interactions among epigenetic risk factors (including air pollution), *MTHFR* polymorphisms, and the risks of developing leukemia. We completed a comprehensive search of 6 databases to find 54 studies (10,033 leukemia cases and 15,835 controls) for *MTHFR* 677, and 43 studies (8,868 cases and 14,301 controls) for *MTHFR* 1298, published from 1999 to 2014. The results revealed that, in European populations; childhood populations; children from Europe, East Asia, and America; and children with acute lymphocytic leukemia (ALL), *MTHFR* 677 polymorphisms (*both* TT and CT types together and individually) are protective, while CC wildtype was leukemogenic. In addition, *MTHFR* 1298 polymorphisms were protective against ALL and acute myeloid leukemia in European children, and in chronic myeloid leukemia in all adults worldwide and American adults. Air pollution played a role in the increased polymorphisms of *MTHFR* 677 genotypes in childhood leukemia.

## INTRODUCTION

Leukemia is one of the most common types of hematological malignancy among children [1] and adults worldwide [2, 3]. The etiology of leukemia remains mostly unknown; however, it is likely the accumulation of genetic and epigenetic alterations induced by adverse gene-environment interactions [4–6]. The role of *methylenetetrahydrofolate reductase* (*MTHFR)* polymorphisms in the development of leukemia has been extensively examined; however, the evidence about its role in leukemogenesis is inconsistent. The MTHFR is a key enzyme in folate metabolism and DNA methylation [7]. MTHFR enzyme converts homocysteine to methionine in the methylation pathways, which helps generation of 1 million new cells and DNA molecules as well as protein and lipid syntheses daily in humans [8, 9]. Two common polymorphisms in *MTHFR*, C677T (rs 1801133) and A1298C (rs 1801131), have been associated with reduced MTHFR enzymatic activity, which leads to hyperhomocysteinemia, aberrant folate metabolism, and DNA hypomethylation [5]. Abnormal folate metabolism has been found to cause DNA translocations and deletions in hematopoietic progenitor cells, triggering the development of leukemia [5, 7, 8].

However, other studies have reported a protective advantage of carrying *MTHFR* variant alleles against leukemia in both children and adults [9, 10]. Leukemias, just like colorectal carcinomas, are derived from rapid proliferations of tissues, which requires faster DNA synthesis and folate metabolism [11]. Therefore, leukemogenesis would be reduced by DNA hypomethylation at the local tissues and reduced MTHFR enzymatic activities at the bone marrows where active leukemogenesis occurs in the carriers of *MTHFR* polymorphisms [6, 8, 12]. Further, evidence has shown that environmental factors and lifestyle behaviors have played pivotal roles in developing leukemia through modulating methylation pathways [13] and alternating folate metabolism [14–16]. For example, many studies have supported the association between childhood leukemia and exposure to air pollutants [17–23]. Outdoor air pollution containing substances such as nitrogen dioxide, benzene, and polycyclic aromatic hydrocarbons, has been classified as carcinogenic [18, 19]. Benzene in particular has potent hematotoxicity and carcinogenicity. Besides linking direct exposure and cancer, studies also reported the increased leukemia risk among children whose mothers had occupational exposure to benzene [13, 17, 24]. Additional studies reported the potential leukemogenesis effect of benzene in children could occur even at ambient air levels lower than currently allowed limits [17, 18]. Despite these findings, previous meta-analyses did not examine the effects of gene-environment interactions, specifically air pollution, on the associations with *MTHFR* polymorphisms and leukemia risk [25–27]. To fill this gap in the evidence [28, 29], we conducted a meta-analysis with meta-predictive techniques to examine the impact of exposure to air pollution on the role of *MTHFR* polymorphism in leukemogenesis of various leukemias across lifespans of children and adults.

## RESULTS

### Characteristics of the studies

**Figure 1:**
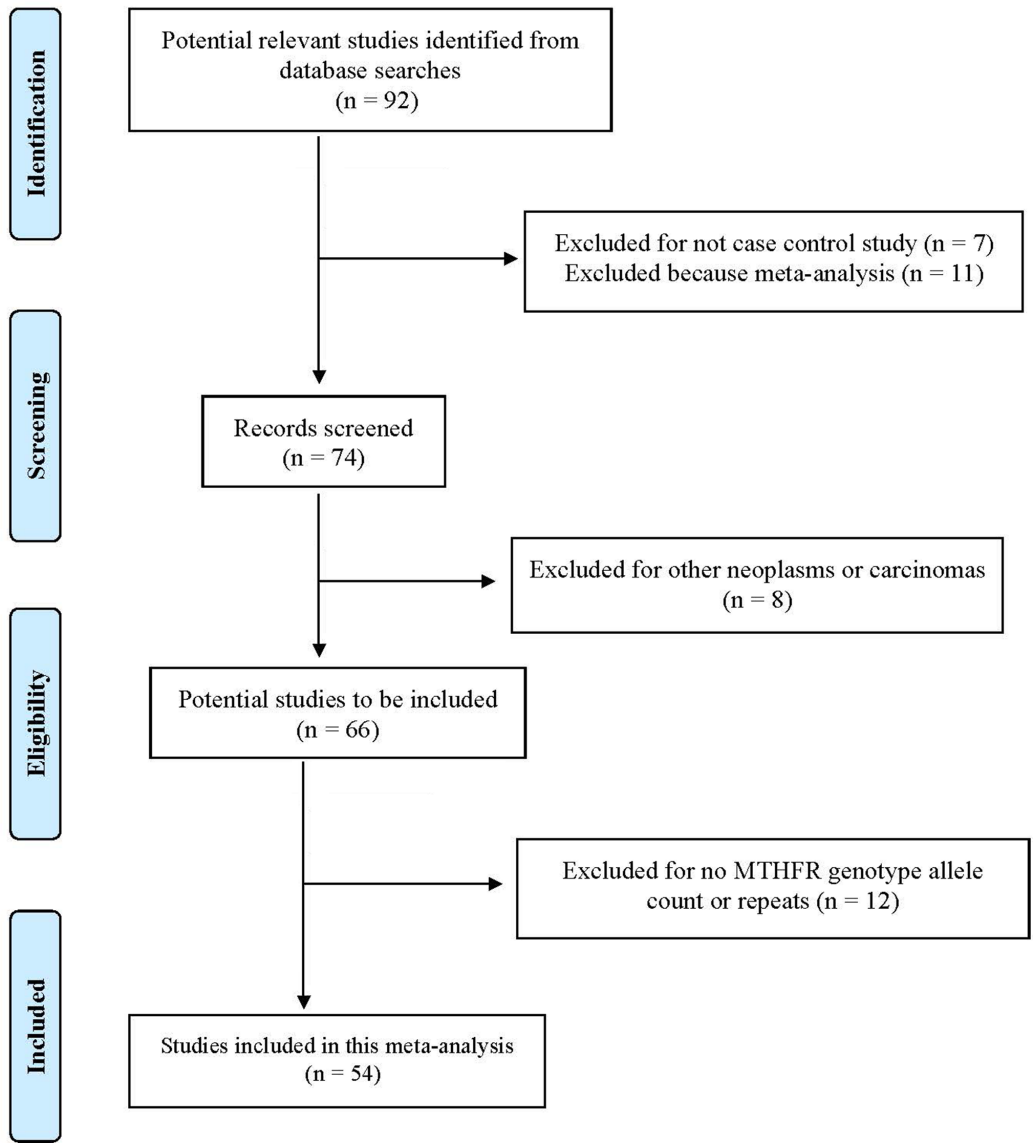
Progression by which studies were selected for this meta-analysis

We have summarized how we selected studies in Figure 1. A total of 92 articles were initially identified between 1965 and August 2016. We located a total of 54 eligible studies (10,033 leukemia cases and 15,835 controls) for *MTHFR* 677 and 43 studies (8,868 leukemia cases and 14,301 controls) for *MTHFR* 1298 polymorphisms. These studies were conducted in five continents, included both childhood and adulthood leukemias, and specified subtypes: acute lymphoblastic leukemia (ALL), acute myeloid leukemia (AML), chronic myeloid leukemia (CML), and various combinations of mixed types (Supplementary Table S1, Supplementary Figure S1).

### Pooled analyses by leukemia types and age groups

For all included study groups, the pooled analysis showed significant associations between *MTHFR* 677 TT and TT plus CT polymorphisms as protective against leukemogenesis in 16 European studies (See Table 1A, and Supplementary Table S2). In addition, for children, *MTHFR* 677 TT and CT polymorphisms together were protective against leukemia (RR = 0.96, 95% confidence interval [CI = 0.93, 0.99], *p* = .0154), while CC wildtype was associated with increased risk of leukemia (RR = 1.04, 95% CI [1.01, 1.08], *p* = .0139) (Table 1A). Subgroup analyses by regions showed that *MTHFR* 677 polymorphisms were protective against leukemia in children from Europe, East Asia, and America (Europe–CT type: RR = 0.94, 95% CI [0.89, 0.99], *p* = .0316; TT+CT types: RR = 0.94, 95% CI [0.90, 0.99], *p* = .0187; East Asia–CT type: RR = 0.93, 95% CI [0.86, 1.00], *p* = .0491; America – TT type: RR = 0.70, 95% CI [0.52, 0.95], *p* = .0212; TT+CT types: RR = 0.83, 95% CI [0.68, 0.99], *p* = .0449) (Table 1A, Table 2). On the other hand, *MTHFR* 677 CC wildtype was a risk genotype for developing leukemia in children from Europe and America (Europe: RR = 1.11, 95% CI [1.02, 1.22], *p* = .0207; America: RR = 1.09, 95% CI [1.02, 1.20], *p* = .0451). Subgroup analyses by leukemia types again demonstrated the protective role of *MTHFR* 677 TT polymorphism against ALL in American children (RR = 0.71, 95% CI [.51, 0.99], *p* = .0405) while MTHFR 677 CC and CT genotypes increased risk of ALL in American children (RR = 1.05, 95% CI [1.00, 1.09], *p* = .0388). The pooled meta-analyses did not show significant roles of *MTHFR* 677 polymorphisms in any of the adulthood leukemias (Table 1A, Table 2).

**Table 1A:** Schema of significant findings across studies on *MTHFR 677* genotypes and risk of leukemia

*NTHFR* 677	All N = 62 Studies (n Case/ n Control) (10,033/15,838)	Children			Adults				
		All children 39 Studies (5,614/8,474)	ALL 35 Studies (5,161/7,796)	ALL+AML 4 Studies (1,331/1,304)	All adults 23 Studies (3,554/6,575)	ALL 19 Studies (3,254/5,827)	AML 2 Studies (82/300)	CML 8 Studies (1,342/1,812)	Mixed 5 Studies (1,226/2,884)
Overall (62 Studies)	NS	Risk Type: CC Protective: TT + CT	NS	NS	NS	NS	NS	NS	NS
**Subgroups**									
European (16 Studies)	16 Studies (3,901/5,767) Risk Type: CC Protective Type: TT & TT+CT	11 Studies (2,660/4,347) Risk Type: CC Protective: CT & CT+TT	9 Studies (1,560/3,387) NS	2 Studies (1,100/960) NS	5 Studies (1,223/1,420) NS	3 Studies (357/481) NS	0 --	2 Studies (884/939) NS	0 --
East Asian (19 Studies)	19 Studies (1,242/2,872) NS	11 Studies (1,365/1,928) Protective: CT	10 Studies (1,198/1,688) NS	1 Study (87/120) NS	8 Studies (938/3,590) NS	4 Studies (421/1,056) NS	1 Study (55/200) NS	1 Study (40/200) NS	2 Studies (422/2,134) NS
South Asian (9 Studies)	9 Studies (1,993/2,022) NS	8 Studies (1,576/1,530) NS	7 Studies (1,073/1,444) NS	0 --	1 Study (528/510) NS	0 --	0 --	0 --	1 Study (417/492) NS
American (9 Studies)	9 Studies (848/1,266) NS	5 Studies (649/793) Risk Type: CC Protective: TT & TT+CT	4 Studies (505/569) Risk Type: CC+CT Protective: TT	1 Study (144/224) NS	4 Studies (199/473) NS	0 --	1 Study (27/100) NS	3 Studies (172/373) NS	0 --
Middle East (7 Studies)	7 Studies (803/821) NS	3 Studies (234/311) NS	3 Studies (234/311) NS	0 --	4 Studies (569/510) NS	1 Study (33/82) NS	0 ---	1 Study (378/258) NS	2 Studies (387/258) NS
African (2 Studies)	2 Studies (185/441) NS	1 Study (88/311) NS	1 Study (88,311) NS	0 --	1 Study (97/130) NS	0 --	0 -	1 Study (97/130) NS	0 --

*Note*:

NS: No Statistical Significance; -- No data;

ALL: Acute Lymphoblastic Leukemia; AML: Acute Myeloid Leukemia; CML: Chronic Myeloid Leukemia.

**Table 1B:** Schema of significant findings across studies on *MTHFR 1298* genotypes and risk of leukemia

*NTHFR* 1298	All N= 50 Studies (n Case/ n Control) (8,368/14,301)	Children			Adults				
		All children 31 Studies (5,614/8,474)	ALL 28 Studies (4,531/7,396)	ALL+AML 4 Studies (1,114/1,326)	All adults 19 Studies (3,157/5,697)	ALL 7 Studies (767/1,537)	AML 2 Studies (88/300)	CML 7 Studies (1,193/1,629)	Mixed 3 Studies (1,151/2,231)
Overall (50 Studies)	NS	NS	NS	NS	NS	NS	NS	Risk Type: AA Protective CC=AC	NS
**Subgroups**									
European (12 Studies)	12 Studies (3,538/4,591) NS	8 Studies (2,349/3,548) NS	6 Studies (1,353/2,626) NS	2 Studies (996/958) Risk Type: AA Protective: CC+AC	4 Studies (1,189/1,367) NS	3 Studies (357/481) NS	0 --	1 Study (836/886) NS	0 --
East Asian (16 Studies)	16 Studies (2,670/5,318) NS	8 Studies (1,194/1,728) NS	7 Studies (1,107/1,608) NS	1 Studies (87/120) NS	8 Studies (1,476/3,590) NS	4 Studies (410/1056) NS	1 Study 55/200 NS	1 Study (40/200) NS	2 Studies (971/2,134) NS
South Asian (6 Studies)	6 Studies (1,210/1,723) NS	6 Studies (1,210/1,723) NS	6 Studies (1,210/1,723) NS	0 --	0 --	0 --	0 --	0 --	0 --
American (9 Studies)	9 Studies (739/1,291) NS	5 Studies (677/769) NS	4 Studies (606/698) NS	1 Studies (71/71) NS	4 Studies (199/473) NS	0 --	1 Study 27/100 NS	3 Studies (172/373) Risk Type: AA Protective: CC+AC	0 --
Mideast (5 Studies)	5 Studies (526/578) NS	3 Studies (233/311) NS	3 Studies (233/311) NS	0 --	2 Studies (293/267) NS	0 --	0 --	1 Study (149/170) NS	1 Study (144/97) NS
African (2 Studies)	2 Studies (185/440) Risk Type: AA Protective: AC, AC+CC	1 Study (88/310) NS	1 Study (88/310) NS	0 --	1 Study (97/130) NS	0 --	0 --	1 Study (97/310) NS	0 --

*Note*:

NS: No Statistical Significance; -- No data;

ALL: Acute Lymphoblastic Leukemia; AML: Acute Myeloid Leukemia; CML: Chronic Myeloid Leukemia.

**Table 2:** Pooled meta-analysis: *MTHFR 677* genotypes and risks of leukemia in children

Genotype by Race or Ethnicity (Number of studies)	Leukemia Case (*N* = 6572) *n* (%)	Control (*N* = 9220) *n* (%)	Test of Heterogeneity			Statistical Model	Test of Association	
			Q	*p*	I^2^		Risk Ratio (95% Cl)	*p*
**TT (39)**	723 (11.01)	1064 (11.54)	50.00	0.0921	24%	Fixed	0.95 (0.87 to 1.04)	0.2902
Europe (11)	284 (10.68)	509 (11.71)	9.13	0.6094	0%	Fixed	0.91 (0.79 to 1.05)	0.1938
America (5)	59 (9.09)	100 (12.61)	2.301	0.6804	0%	Fixed	0.70 (0.52 to 0.95)	0.0212
Middle East (3)	20 (8.55)	19 (6.11)	1.361	0.5064	0%	Fixed	1.40 (0.76 to 2.59)	0.2800
South Asia (8)	86 (5.46)	66 (4.31)	2.757	0.8387	0%	Fixed	0.90 (0.63 to 1.27)	0.5411
East Asia (11)	267 (19.56)	350 (25.64)	25.88	0.0039	61.4%	Random	1.14 (0.87 to 1.50)	0.3306
Africa (1)	7 (7.95)	20 (6.43)					1.24	
**CT (39)**	2688 (40.91)	3921 (42.53)	76.89	0.0003	49.3%	Random	0.95 (0.90 to 1.01)	0.1219
Europe (11)	1119 (42.07)	1953 (44.93)	16.93	0.1101	35%	Fixed	0.94 (0.89 to 0.99)	0.0316
America (5)	264 (40.68)	354 (44.64)	8.791	0.0665	54.5%	Fixed	0.91 (0.81 to 1.03)	0.1434
Middle East (3)	100 (42.74)	126 (40.51)	4.185	0.1234	52.2%	Fixed	1.05 (0.86 to 1.28)	0.6289
South Asia (8)	568 (36.04)	447 (29.22)	21.73	0.0028	67.8%	Random	1.12 (0.90 to 1.38)	0.3090
East Asia (11)	595 (43.59)	906 (66.37)	18.26	0.0507	45.2%	Fixed	0.93 (0.86 to 1.00)	0.0491
Africa (1)	42 (47.73)	135 (43.41)					1.10	
**CC (39)**	3161 (48.10)	4235 (45.93)	95.06	<0.0001	59%	Random	1.04 (1.01 to 1.08)	0.0139
Europe (11)	1257 (47.26)	1885 (43.26)	22.85	0.0113	56.2%	Random	1.11 (1.02 to 1.22)	0.0207
America (5)	326 (50.23)	339 (42.75)	10.33	0.0352	61.3%	Random	1.09 (1.02 to 1.20)	0.0451
Middle East (3)	114( 48.72)	166 (53.38)	3.135	0.2086	36.2%	Fixed	0.92 (0.77 to 1.08)	0.3047
South Asia (8)	922 (58.50)	1017 (66.47)	25.76	0.0006	72.8%	Random	0.95 (0.86 to 1.06)	0.3603
East Asia (11)	503 (36.85)	672 (49.23)	12.59	0.2476	20.6%	Fixed	1.07 (0.97 to 1.17)	0.1735
Africa (1)	39 (44.32)	156 (50.16)					0.88	
**TT + CT (39)**	3411 (51.90)	4985 (54.07)	81.99	< 0.0001	53.7%	Random	0.96 (0.93 to 0.99)	0.0154
Europe (11)	1403 (52.74)	2462 (56.64)	18.89	0.0417	47.1%	Random	0.94 (0.90to 0.99)	0.0187
America (5)	323 (49.77)	454 (57.25)	12.35	0.0149	67.6%	Random	0.83 (0.68 to 0.99)	0.0449
Middle East (3)	120 (51.28)	145 (46.62)	3.36	0.1867	40.4%	Fixed	1.09 (0.92 to 1.30)	0.3
South Asia (8)	654 (41.49)	513 (33.53)	25.60	0.0006	72.7%	Random	1.10 (0.89to 1.36)	0.3572
East Asia (11)	862 (63.15)	1256 (65.15)	11.60	0.3126	13.8%	Fixed	0.97 (0.92 to 1.02)	0.1761
Africa (1)	49 (55.68)	155 (49.84)					1.12	
**CC+CT (39)**	5849 (88.99)	8156 (88.46)	6.50	> 0.9999	0%	Fixed	1.34 (0.85 to 2.10)	0.2039
*TT + CT Risk < 1*								
TT (14)	229 (9.31)	385 (11.47)	9.77	0.7124	0%	Fixed	0.81 (0.69 to 0.95)	0.0101
CT (14)	971 (39.46)	1496 (44.55)	16.84	0.2067	22.8%	Fixed	0.89 (0.84 to 0.95)	0.0005
CC (14)	1261 (51.24)	1477 (43.98	25.9	0.0175	49.8%	Random	1.21 (1.11 to 1.32)	<0.0001
TT+CT (14)	1200 (48.76)	1881 (56.02)	25.42	0.0203	48.9%	Random	0.85 (0.78 to 0.92)	<0.0001
CC+CT (14)	2232 (90.69)	2973 (88.53)	11.37	0.5795	0%	Fixed	1.02 (1.01 to 1.04)	0.0078
*TT+CT Risk >1*								
TT (8)	69 (6.71)	113 (6.34)	3.77	0.7077	0%	Fixed	0.96 (0.72 (1.27)	0.7562
CT (8)	396 (38.48)	562 (31.54)	7.25	0.4033	3.5%	Fixed	1.19 (1.08 to 1.32)	0.0008
CC (8)	564 (54.81)	1107 (62.12)	10.06	0.1854	30.4%	Fixed	0.91 (0.85 to 0.97)	0.0023
TT+CT (8)	465 (45.19)	675 (37.88)	9.08	0.247	22.9%	Fixed	1.15 (1.05 to 1.26)	0.0016
CC+CT (8)	960 (93.29)	1669 (93.66	3.96	0.6817	0%	Fixed	1.00 (0.98o 1.03)	0.7582
*TT+CT Risk~ 1*								
TT (17)	425 (13.79)	566 (13.87)	29.36	0.0216	45.5%	Random	1.10 (0.91 to 1.34)	0.3211
CT (17)	1321 (42.86)	1863 (45.66)	29.00	0.0239	44.8%	Random	0.95 (0.87 to 1.03)	0.2083
CC (17)	1336 (43.35)	1651 (40.47)	24.33	0.0826	34.2%	Fixed	1.02 (0.97 to 1.08)	0.4464
TT+CT (17)	1746 (56.65)	2429 (59.53)	21.48	0.1608	25.5%	Fixed	0.98 (0.94 to 1.030)	0.4546
CC+CT (17)	2657 (86.21)	3514 (86.13)	37.05	0.0021	56.8%	Random	0.99 (0.96 to 1.02)	0.4914

*Note*:

*MTHFR* = methylenetetrahydrofolate reductase; CI = confidence interval; TT = MTHFR homozygous genotype TT; CC = MTHFR homozygous genotype CC; CT = MTHFR homozygous genotype CT; TT+CT = MTHFR homozygous genotype TT plus CT; CC+CT = MTHFR homozygous genotype CC plus CT.

*European countries: Greece, Germany, Italy, Netherlands, Serbia, Slovenia, and United Kingdom; American countries: Canada, Brazil; Middle Eastern countries: Iran, Turkey; Asian countries: East Asia: China, South Korea and Taiwan, South Asia: Singapore, Philippines, Indonesia and India; African country: Egypt*.

*TT+CT Risk > 1 countries: Canada, Taiwan, Philippines, India, Iran, and Egypt*.

*TT+CT Risk < 1 countries: Serbia, Slovenia, Greece, Netherlands, United Kingdom, Portugal, Brazil, and Singapore*.

*TT+CT Risk ~ 1 countries: Germany, South Korea, China, Indonesia, and Turkey*.

Similar to *MTHFR* 677, *MTHFR* 1298 CC and AC polymorphisms played protective roles against ALL and AML in European children, cases aggregated, in 4 studies (RR = 0.89, 95% CI [.82, .98], *p* = .0124), while the AA wildtype was associated with increased risk of ALL and AML, cases aggregated, in the same studies (RR =1.12, 95% CI [1.03, 1.23], *p* = .0126) (Table 1B, Supplementary Table S3). Similarly, in 7 studies conducted worldwide that included adults, *MTHFR* 1298 CC and AC polymorphisms were protective against CML (RR = 0.82, 95% CI [0.66, 1.01], *p* = .0421) while AA wildtype was a risk type for developing CML (worldwide, 7 studies: RR =1.14, 95% CI [1.00, 1.31], *p* = .0477; America, 3 studies: RR =1.18, 95% CI [1.01, 1.38], *p* = .0416) (Table 1B). The results from both funnel plot and Egger's linear regression test showed no significant publication bias in any eligible studies.

### Subgroup analyses by countries

**Figure 2:**
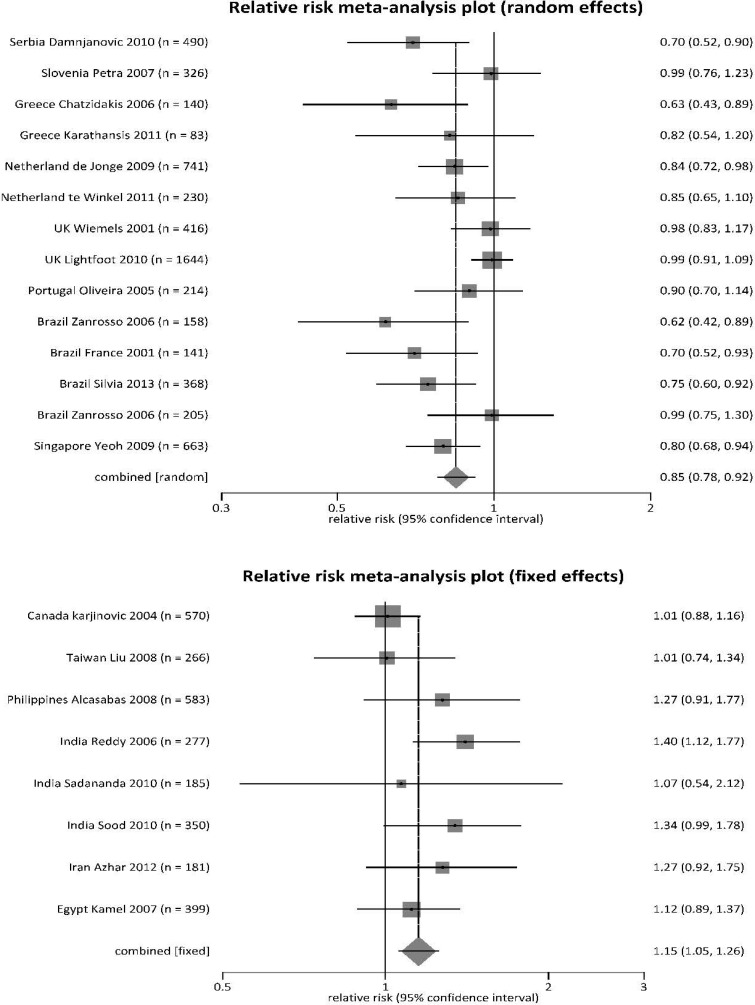

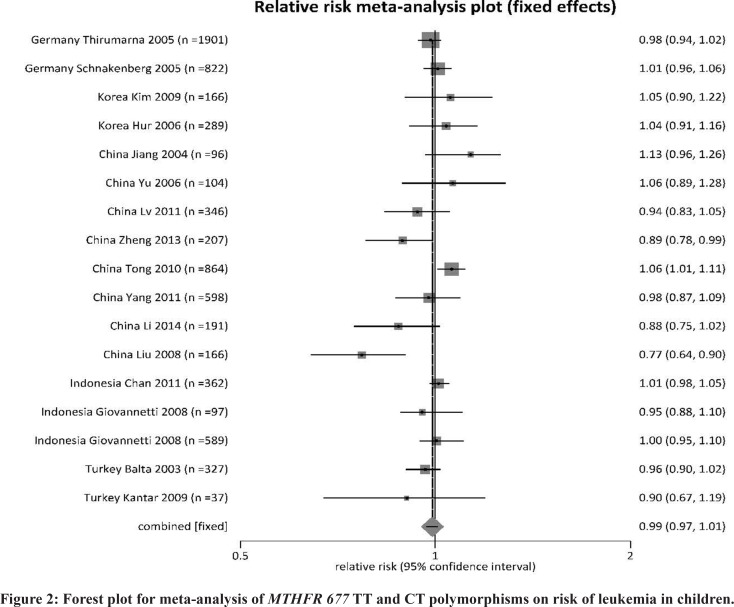
Forest plot for meta-analysis of *MTHFR 677* TT and CT polymorphisms on risk of leukemia in children

To identify sources of heterogeneity, we further analyzed subgroups of countries, grouping them by risk for childhood leukemias (Table 2). We divided countries based on *MTHFR* 677 polymorphisms (TT plus CT together) being protective (RR < 1), risks (RR > 1), or mixed effects (RR varied around 1). The countries that had *MTHFR* polymorphisms as protective genotypes (RR < 1) included mostly European countries (Serbia, Slovenia, Greece, Netherlands, United Kingdom [UK], Portugal), plus Brazil and Singapore (Figure 2A). The countries that had *MTHFR* polymorphisms as risk genotypes (RR > 1) included Canada, Taiwan, the Philippines, India, Iran and Egypt (Figure 2B). With mixed effects, *MTHFR* 677 polymorphisms had varied effects on development of childhood leukemias in Germany, South Korea, China, Indonesia and Turkey (Figure 2C).

To validate the heterogeneous findings, we further utilized a geographic information system (GIS) to visualize regional distributions [30]. The global maps demonstrated the variations in the distribution of *MTHFR* 677 polymorphisms and their inconsistent roles in leukemogenesis in children across regions (Supplementary Figures S2, S3, and S4). In the first two GIS maps, we used the continuous color spectrum from yellow to red, representing the increasing levels of polymorphisms, and, in the third map, red-green colors—red indicating leukemia risk, and green indicating protective effects. Similar to the pooled meta-analysis, GIS maps showed that *MTHFR* 677 polymorphisms individually (CT, TT) and together (TT and CT) played a protective role against childhood leukemia in most countries except Taiwan, China, India, Turkey, and Egypt (Supplementary Figures S2, S3, S4).

### Meta-prediction

Given the role of heterogeneous findings on the effects of *MTHFR* polymorphisms, we performed meta-predictive analyses using both big-data analytics and conventional analyses on childhood leukemias (Table 3). We used both partition tree and Turkey's tests to examine the potential interaction between air pollution and polymorphisms, and their combined impact on leukemia risk. Based on guidelines from the World Health Organization [32] on air quality measures, we used the death from air pollution (AP death) as the measure for air quality (Level 2 = 50–100 or less annual deaths/million population; Level 3 = 100–250 deaths/million; and Level 4 = 250–400 or greater deaths/million) [33]. The partition tree and Tukey's tests showed significant differences between groups exposed to low versus high air pollution on the percentage of *MTHFR* 677 TT polymorphism in childhood leukemias (mean difference = + 8.7%, 95% CI [9%, 16.6%], *p* = .0258). To further illustrate this significance, we plotted those results on nonlinear curves. With the increased air pollution rates from low (Level 2) to high (Level 4), there was a steeper increase in the percentages of *MTHFR* 677 TT polymorphisms in the leukemia-case groups than in control groups (Supplementary Figure S5). The heat map also demonstrated dense distribution of the *MTHFR* 677 TT mutation in leukemia-case groups (more red blocks) than in control groups (Supplementary Figure S6).

**Table 3:** Meta-prediction: Air pollution associated death (AP Death) on MTHFR 677 genotypes for controls (ct) and leukemia cases (ca) in children

Variable	Partition tree						Tukey's test				
	AICc	AP Death	Count	Mean	SD	Level Compared	Difference	SE Difference	Lower CI	Upper CI	*p*
TT%ct	−100.35	2 & 3	21	0.096	0.063	4/2	0.045	0.028	−0.024	0.115	0.2605
		4	18	0.126	0.063	4/3	0.026	0.023	−0.083	0.083	0.5104
						3/2	0.019	0.029	−0.054	0.093	0.7936
TT%ca	−91.168	2 & 3	21	0.076	0.045	4/2	0.087	0.032	0.009	0.166	0.0258
		4	18	0.143	0.092	4/3	0.054	0.026	−0.010	0.118	0.1094
						3/2	0.033	0.034	−0.049	0.116	0.5910
CT%ct	−63.886	2 & 3	21	0.389	0.113	4/2	0.074	0.046	−0.037	0.186	0.2476
		4	18	0.452	0.083	4/3	0.063	0.037	−0.029	0.154	0.2262
						3/2	0.012	0.048	−0.106	0.129	0.9686
CT%ca	−63.295	2	8	0.365	0.121	3/2	0.040	0.049	−0.080	0.159	0.6995
		3 & 4	31	0.401	0.096	4/2	0.031	0.046	−0.083	0.144	0.7842
						3/4	0.009	0.038	−0.084	0.101	0.9719
CC%ct	−35.288	4	18	0.422	0.115	2/4	0.120	0.066	−0.041	0.280	0.1762
		2 & 3	21	0.515	0.166	3/4	0.089	0.054	−0.042	0.220	0.2367
						2/3	0.031	0.069	−0.138	0.200	0.8950
CC%ca	−41.963	3 & 4	31	0.479	0.132	2/4	0.118	0.060	−0.029	0.266	0.1360
		2	8	0.578	0.138	2/3	0.073	0.063	−0.082	0.228	0.4926
						3/4	0.046	0.049	−0.075	0.166	0.6265
RRTT	66.809	2 & 3	19	0.832	0.286	4/2	0.399	0.270	−0.263	1.062	0.3134
		4	18	1.224	0.748	4/3	0.357	0.213	−0.167	0.881	0.2312
						3/2	0.043	0.286	−0.659	0.745	0.9878
RRCT	1.202	4	18	0.904	0.241	3/4	0.146	0.085	−0.062	0.354	0.2129
		2 & 3	21	1.025	0.223	2/4	0.107	0.104	−0.148	0.362	0.5655
						3/2	0.040	0.110	−0.229	0.308	0.9317
RRCC	14.865	3 & 4	31	1.079	0.257	4/3	0.139	0.099	−0.102	0.381	0.3466
		2	8	1.176	0.345	2/3	0.137	0.127	−0.174	0.449	0.5339
						4/2	0.002	0.121	−0.294	0.298	0.9998

*Note. MTHFR* = methylenetetrahydrofolate reductase; CI = confidence interval; AICc = Akaike's information criterion correction; AP Death = annual death rate from air pollution, in levels per million people ( 2 = 50–100 deaths, 3 = 100–250 deaths, 4 = 250–400+ deaths); RR = Risk Ratio; ct = controls; ca = Leukemia cases; TT%ct = the percentages of TT mutations in the control group; TT%ca = the percentages of TT mutations in the Leukemia cases; CT%ct = the percentages of CT mutations in the control group; CT%ca = the percentages of CT mutations in the Leukemia cases; CC%ct = the percentages of CC genotype in the control group; CC%ca = the percentages of CC genotype in the Leukemia cases; RRTT = risk ratio of TT mutation between case and control groups; RRCT = risk ratio of CT mutation between case and control groups; RRCC = risk ratio of CC genotype between case and control groups.

## DISCUSSION

To date, many studies have investigated the association between *MTHFR* polymorphisms and leukemias; however, results have been inconsistent. Consistent with previous analyses [4, 12, 27, 34–36], our comprehensive analysis generated from studies to date indicated significant heterogeneity in the distributions of *MTHFR* polymorphisms across age groups, regions, and types of leukemia. We found *MTHFR* 677 TT and CT polymorphisms played protective roles, while the CC wildtype was a risk genotype for leukemia in all child populations worldwide combined, as well as in children from Europe, American, and East Asia. In addition, *MTHFR* 1298 polymorphisms were protective against ALL and AML in children from Europe, as well as CML in adults. Our analyses by countries in subgroups showed that *MTHFR* 677 polymorphisms were protective mainly in European countries but leukemogenic in South Asian and African countries. Such variations among world populations might be associated with different underpinning mechanisms and pathways for the effects of *MTHFR* 677 polymorphisms in childhood leukemia in relation to geographic locations [5, 7, 8].

Furthermore, using meta-predictive techniques, we showed a potential impact of air pollution on the link between *MTHFR* gene polymorphisms and leukemogenesis. In countries with high levels of air pollution (level 4), MTHFR 677 polymorphisms played a protective role in European countries (i.e., UK, Greece, Portugal), had mixed effects in China and Turkey, and posed a risk for childhood leukemia in Taiwan and Iran. Among countries with low levels of air pollution (level 2), *MTHFR* 677 polymorphisms played protective roles against childhood leukemia in Brazil but were leukemogenic in India. These findings illustrate the complexity of gene-environment interactions across regions and ethnic groups. Air pollution has direct [18, 19] and indirect effects [13, 17, 24] on the development of childhood leukemia, possibly aggravated more in warmer regions. Given that we do not yet have sufficient studies, especially in regions other than Europe and China, further studies are warranted to examine the mechanisms of *MTHFR* polymorphisms in leukemias. In summary, research should study how genes, environment, and additional risk factors including air pollution interact with *MTHFR* polymorphisms; such studies across different regions and ethnicities will advance the prevention of leukemia, especially for children.

## MATERIALS AND METHODS

### Study search strategy

We followed the guidelines for Preferred Reporting Items of Systematic Reviews and Meta-Analysis (PRISMA) [37], and conducted a comprehensive literature search from databases of MEDLINE (PubMed), Cochrane Library, Embase, e-Books, EBSCO, and PsychoInfo from 1965 to August, 2016. The search strategy included the following keywords and terms: “MTHFR gene” or “leukemia” and “adult“ or “children,” “environment,” “diet prevention,” “folate,” “lifestyle,” “behavior,” “case control design” and “meta-analysis,” and all leukemia types. Additionally, previous meta-analysis and review papers were used to cross check and trace back all original studies. Various databases were searched repeatedly at three different times at least 3 months apart until no additional eligible papers were identified.

### Inclusion/exclusion criteria

The inclusion criteria were that the articles 1) examined the association of the *MTHFR* polymorphisms (C677T and/or A1298C) and leukemia risk using a case-control design, 2) included the genotype frequency in both case and control groups, 3) were written in English or 4) were written in non-English but provided tables with genotype allele frequencies for both case and control groups. Articles were excluded if they 1) were written in non-English without tables listing genotype allele counts, 2) were not case-control studies, or 3) had missing counts of genotype frequency.

Based on those criteria, we first identified 92 articles. We excluded 38 articles, including 11 meta-analyses, 7 non-case-control studies, 8 studies on other neoplasms or carcinomas, and 12 studies that lacked genotype allele counts or duplicates. The remaining 54 papers were included in the final analyses (Figure 1). Two raters independently conducted the process of literature search, screening, data collection, and extraction based on the study protocol. The discrepancy between raters was reduced to zero prior to data analyses.

### Quality assessment

We assessed the quality of these studies [38] based on the criteria from various sources including QUOROM and PRISMA guidelines, and standards for related human genome studies [37, 39]. We assessed domains that included external validity for grouping data (score range of 0–11), internal validity for DNA analysis process and procedural rigor (score range of 0–12), and the quality of detailed reporting on limiting bias (score range of 0–7). Thus, the ranges for each possible total score were from 0 to 30. All included studies showed quality scores from 15 to 22; thus, all 54 studies scored in the top 50% of our range, so we judged their findings trustworthy [39].

### Data synthesis and analysis

We entered data into Excel, and analyzed data using StatsDirect Version 3 updated software (Cheshire, UK). We calculated pooled risk ratios (RR), odds ratios (OR), and 95% CI for the MTHFR subtypes, distinguishing between cases and controls, to test for the associations of genotypes with leukemia. We compared pooled standardized RRs versus ORs to check the differences between the two ratios and found RRs more conservative with less significant findings; therefore, we used RRs in this report.

We used SAS JMP^®^ pro 13 program (SAS Institute, New York, NY, 2016) for meta-prediction analyses to examine how AP death was related to the *MTHFR* polymorphisms and leukemia risk [40]. In addition to conventional analyses, we used big-data analytics including partition trees for these grouping analyses. The goodness of fit was judged by the Akaike's information criterion correction (AICc), with a smaller AIC suggesting a better model [41–43]. To produce more accurate meta-prediction, we used multiple methods to triangulate and cross-validate the findings—including nonlinear fit modeling, partition trees, heat maps, one-way ANOVA, and Tukey's posthoc tests. To compare AICc results with the partition trees, we used Tukey's tests [44]. All *p* values were two-tailed with a significance level at .05. We used the GIS maps to demonstrate the distributions of mutations and their associations with leukemia risks on the global maps [30, 45].

We used Hardy-Weinberg Equilibrium (HWE) analysis to detect the discrepancies in genotype distributions [46, 47]. HWE results were significant in five studies (*p* < 0.05). We performed subgroup analyses on those five studies versus those that had acceptable HWE. However, the results did not yield significant differences. Therefore, all studies regardless of HWE status were included in the final meta-analysis (Supplementary Table S1). Egger's test and funnel plots were performed to detect publication bias [48, 49].

## SUPPLEMENTARY MATERIALS FIGURES AND TABLES








