# Supplementary material for: Meta-prediction of MTHFR gene polymorphism-mutations, air pollution, and risks of leukemia among world populations
Source: Oncotarget. 2016 Dec 10;8(3):4387–98. doi: 10.18632/oncotarget.13876 (PMC5354840; doi:10.18632/oncotarget.13876)
Supplement: Supplementary file 1 [file oncotarget-08-4387-s001.pdf]

Meta-prediction of MTHFR gene polymorphism-mutations, air pollution, and risks of leukemia among world populations

Supplementary Materials

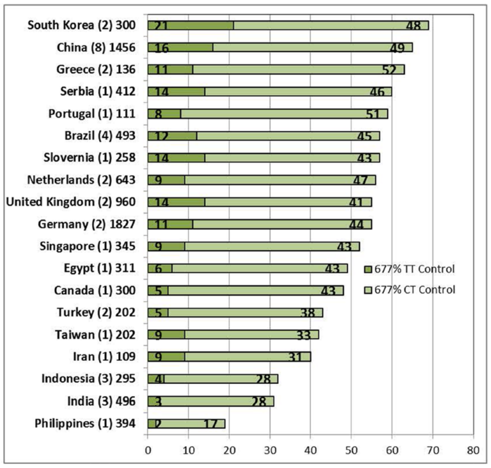

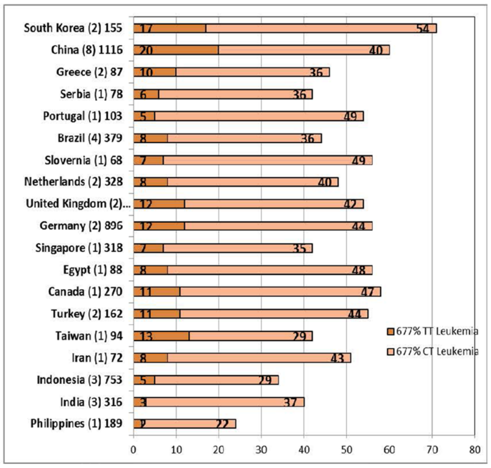

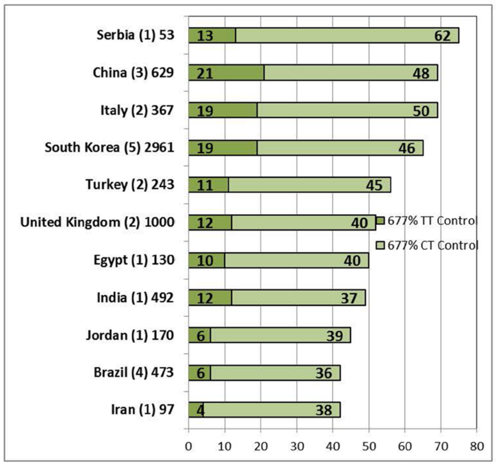

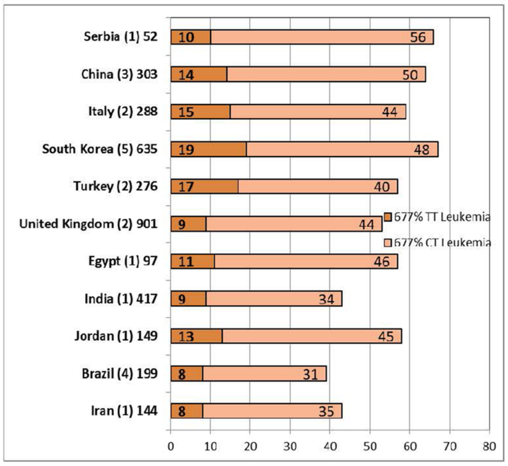

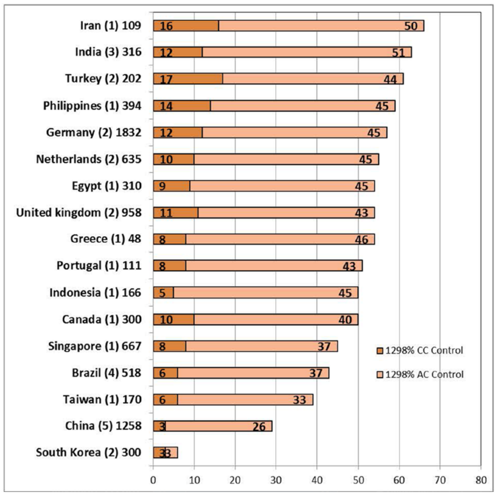

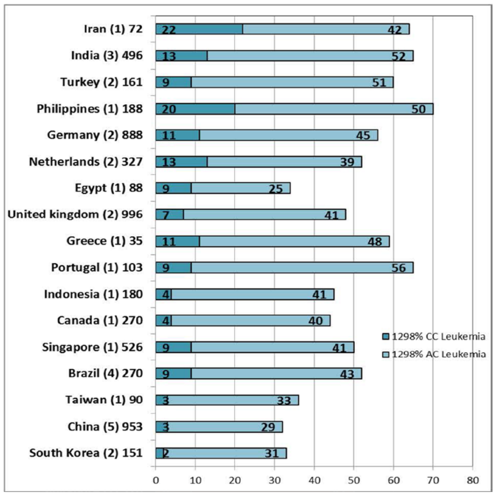

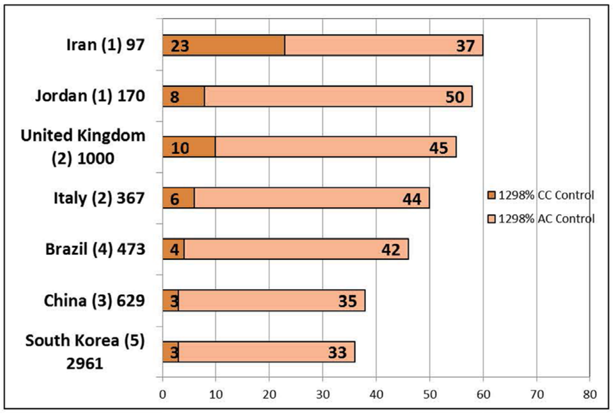

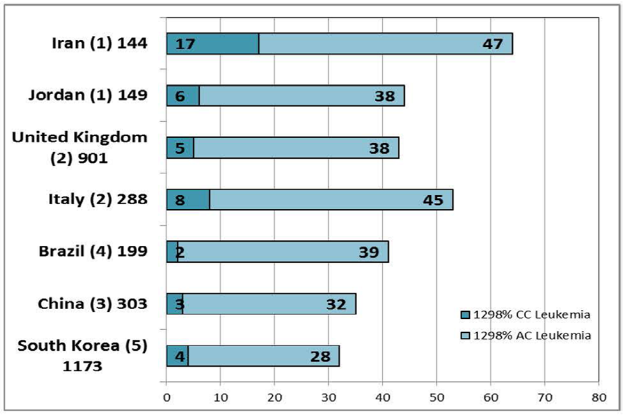

Note:
(a and b) Left column shows country name, number of studies, and number of cases.
677% TT = the percentages of MTHFR 677 TT mutations; 677% CT = the percentages of MTHFR 677 CT mutations.
(c) Left column shows country name, number of studies, and number of cases.
1298 CC% = the percentages of MTHFR 1298 CC mutations; 1298 AC% = the percentages of MTHFR 1298 AC mutations.
(d) Left column shows country name, number of studies, and number of cases.
1298 CC% = the percentages of MTHFR 1298 genotype CC mutations; 1298 AC% = the percentages of MTHFR 1298 AC mutations.

Supplementary Figure S1: MTHFR polymorphism distributions per age groups.

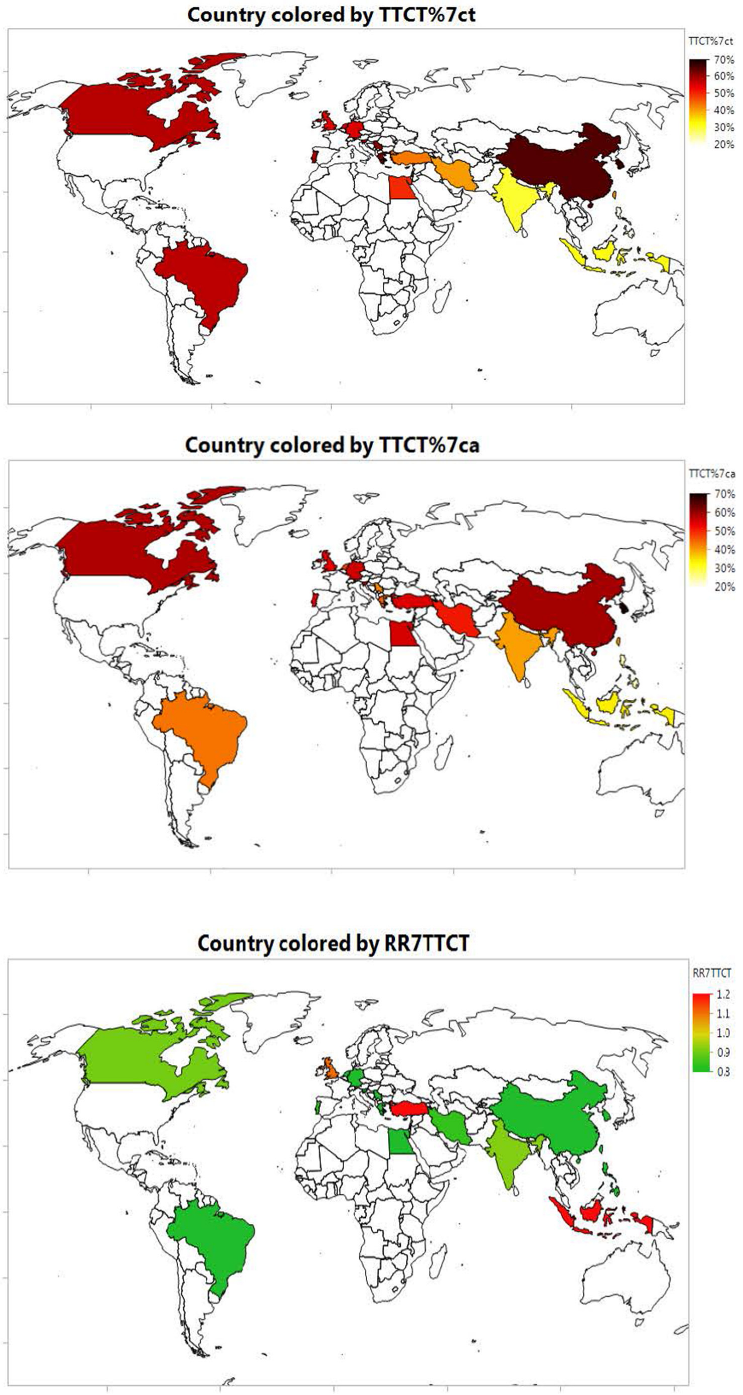

Note: TTCT%7ct = the percentages of MTHFR 677 TT plus CT mutations in control group; TTCT%7ca = the percentages of TT plus CT mutations in the leukemia cases; RR7TTCT = the risk ratio of 677 TT plus CT.

Supplementary Figure S2: GIS map for *MTHFR* 677TTCT in children.

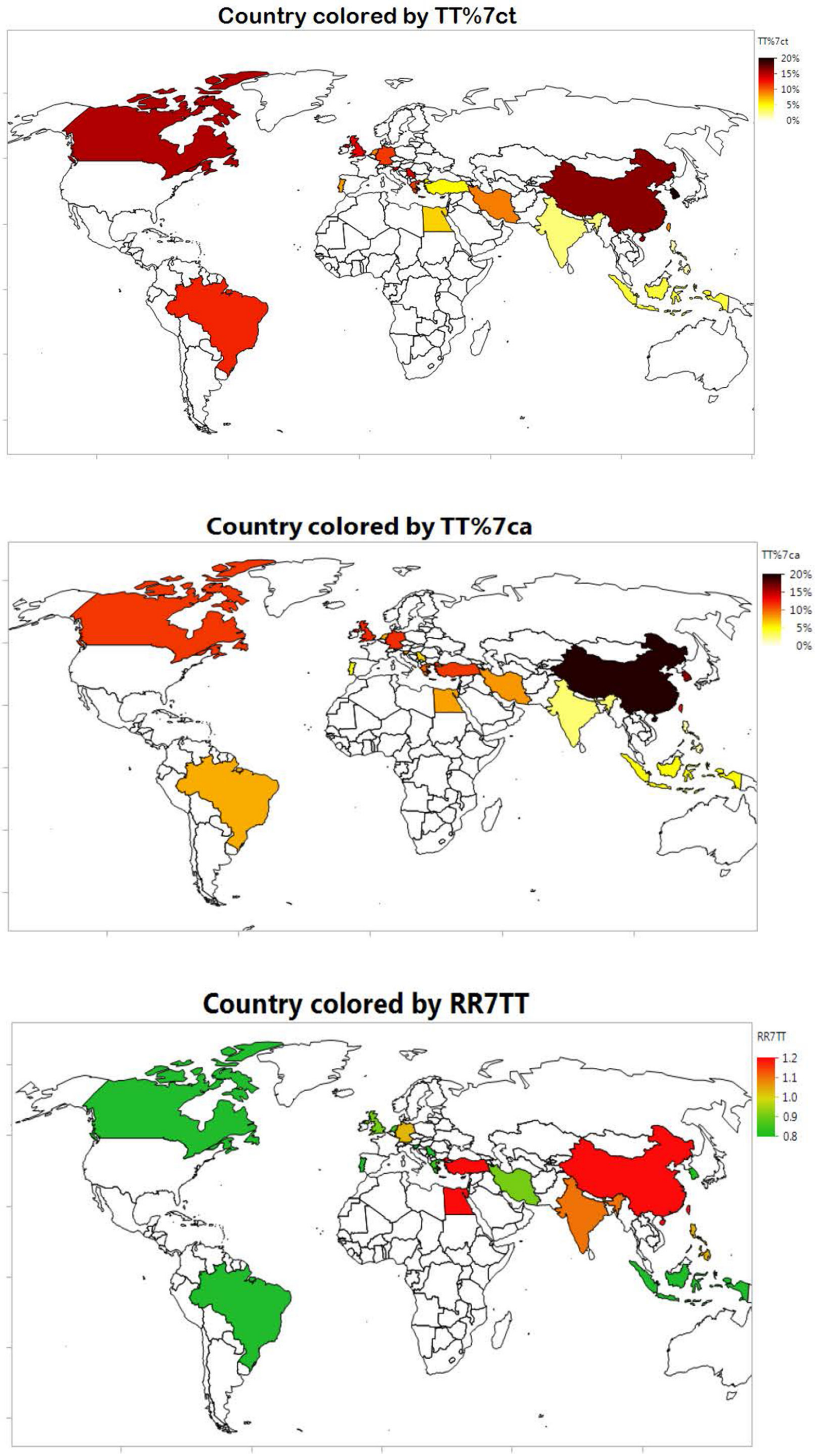

Note: TTCT%7ct = the percentages of MTHFR 677 TT mutation in control group; TTCT%7ca = the percentages of TT mutations in the leukemia cases; RR7TT = the risk ratio of 677 TT.

Supplementary Figure S3: GIS map for *MTHFR* 677TT in children.

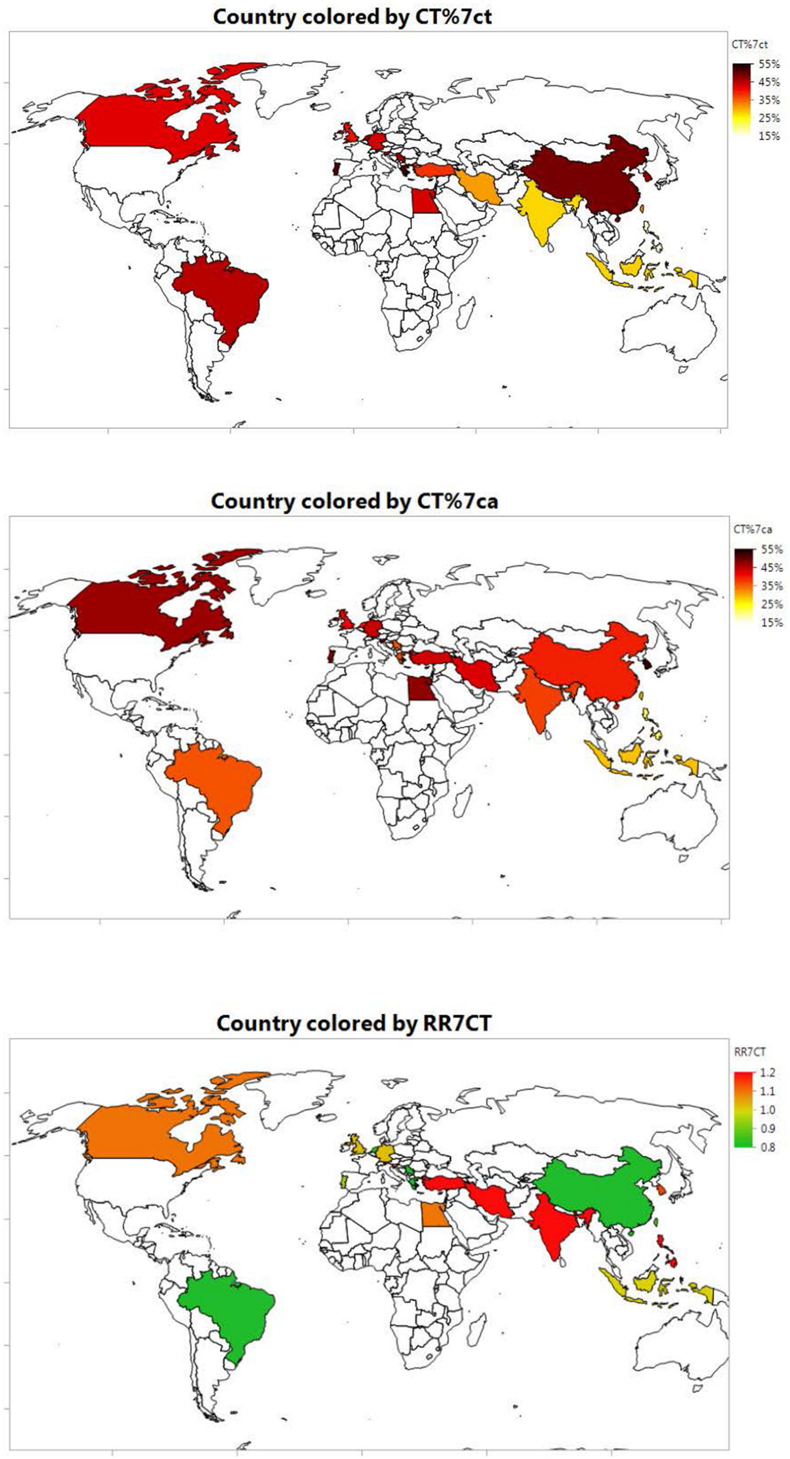

Note: CT%7ct = the percentages of MTHFR 677 CT mutation in control group; CT%7ca = the percentages of CT mutations in the leukemia cases; RR7CT = the risk ratio of 677 CT.

Supplementary Figure S4: GIS map for *MTHFR* 677CT in children.

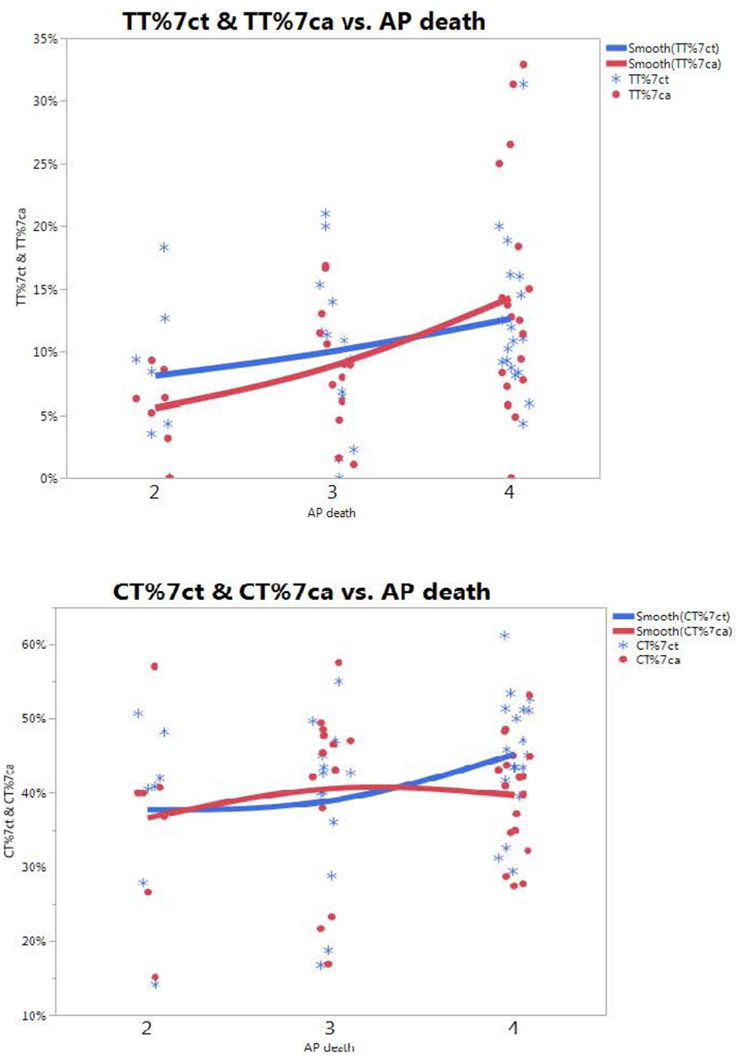

Note: TT%7ct = the percentages of MTHFR 677 TT mutation in control group; TT%7ca = the percentages of CC mutations in the leukemia cases; CT%7ct = the percentages of MTHFR 677 CT mutation in control group; CT%7ca = the percentages of CT mutations in the leukemia cases.

Countries with level 4 AP death rate: Greece, United Kingdom, Portugal, Taiwan, China, Singapore, Iran, Turkey.

Countries with level 3 AP death rate: Slovenia, Netherlands, Germany, Canada, South Korea, Philippine, Indonesia, Egypt.

Countries with level 2 AP death rate: Brazil, India.

Supplementary Figure S5: Nonlinear fit of *MTHFR* 677 TT and CT polymorphisms with AP death in children.

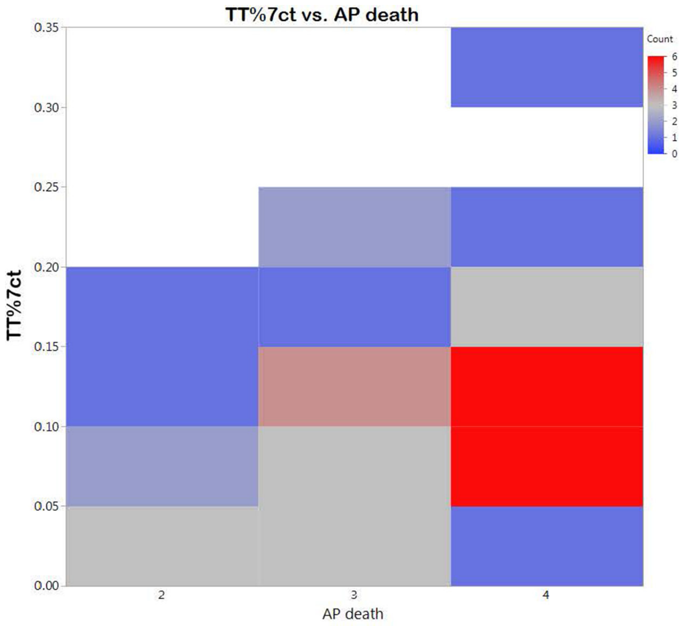

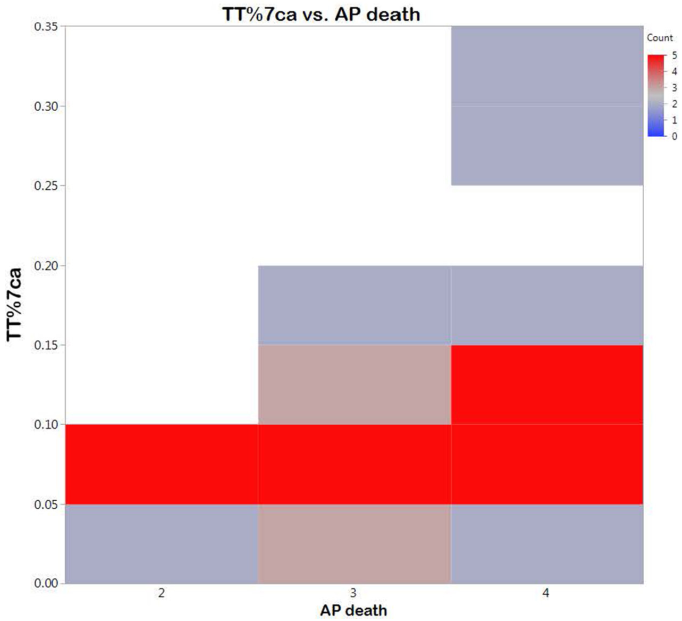

Note: TT%7ct = the percentages of MTHFR 677 TT mutation in control group; TT%7ca = the percentages of CC mutations in the leukemia cases; AP death: Death rates from air pollution, in levels increasing from 2 to 4.

Countries with level 4 AP death rate: Greece, United Kingdom, Portugal, Taiwan, China, Singapore, Iran, Turkey.

Countries with level 3 AP death rate: Slovenia, Netherlands, Germany, Canada, South Korea, Philippine, Indonesia, Egypt.

Countries with level 2 AP death rate: Brazil, India.

Supplementary Figure S6: Heat maps of *MTHFR* 677TT versus AP death in Children.

Supplementary Table S1: Characteristics of studies included in the meta-analysis by continents in the world (62 study groups for *MTHFR* 677, 50 study groups for *MTHFR* 1298). See Supplementary_Table_S1

Supplementary Table S2: Pooled analysis: *MTHFR* 677 genotypes and risks of leukemia for (62 study groups). See Supplementary_Table_S2

Supplementary Table S3: Pooled analysis: *MTHFR* 1298 genotypes and risk of leukemia for (50 study groups). See Supplementary_Table_S3
