# Supplementary material for: Meta-prediction of MTHFR gene polymorphism-mutations, air pollution, and risks of leukemia among world populations
Source: Oncotarget. 2016 Dec 10;8(3):4387–98. doi: 10.18632/oncotarget.13876 (PMC5354840; doi:10.18632/oncotarget.13876)
Supplement: Supplementary file 2 [file oncotarget-08-4387-s002.doc]

**Table S1. Characteristics of studies included in the meta-analysis by continents in the world (62 study groups for *MTHFR* 677, 50 study groups for *MTHFR* 1298).**

| First Author  (Reference number) | Year | Ethnicity  -Country | Group | *MTHFR* 677 | | | | | | | | | | | | *MTHFR* 1298 | | | | | | | | | | | | | | | Quality Score |
| --- | --- | --- | --- | --- | --- | --- | --- | --- | --- | --- | --- | --- | --- | --- | --- | --- | --- | --- | --- | --- | --- | --- | --- | --- | --- | --- | --- | --- | --- | --- | --- |
|  |  |  |  | Cases, Cancer Site (CS), n (%) | | | | | | | Controls, n (%) | | | | | Cases n (%) | | | | | Controls n (%) | | | | | | | | | |  |
|  |  |  |  | LT | | CT | | TT | | CC | CT | TT | CC | HWE | | AA | | CC | | AC | | | AA | | CC | | AC | HWE | | |  |
| **European** | | | | | | | | | | | | | | | | | | | | | | | | | | | | | | | |
| Chatzidakis (1) | 2006 | White  -Greece | C | 1 | 18  (34.6) | | 3  (5.8) | | 31  (59.6) | | 47  (53.4) | 9  (10.2) | 32  (36.4) | | Yes | NA | | | | | | | | | | | | | | | 16  (5,5,6) |
| Karathanasis (2) | 2011 | White  -Greece | C | 1 | 13  (37.1) | | 5  (14.3) | | 17  (48.6) | | 24  (50.0) | 6  (12.5) | 18  (37.5) | | Yes | 14  (40.0) | 4  (11.4) | | 17  (48.6) | | | 22  (45.8) | | 4  (8.3) | | 22  (45.8) | | | Yes | | 21  (6,9,6) |
| Damnjanovic (3) | 2010 | White  -Serbia | C | 1 | 28  (35.9) | | 5  (6.4) | | 45  (57.7) | | 190  (46.1) | 59  (14.3) | 163  (39.6) | | Yes | NA | | | | | | | | | | | | | | | 16  (4,6,6) |
| Jakovljevic  (4) | 2012 | Caucasian  -Serbia | A | 4 | 29  (55.7) | | 5  (9.6) | | 18  (34.6) | | 33  (62.3) | 7  (13.2) | 13  (24.5) | |  | NA | | | | | | | | | | | | | | |  |
| Petra  (5) | 2007 | White  -Slovenia | C | 1 | 33  (48.5) | | 5  (7.4) | | 30  (44.1) | | 110  (42.6) | 36  (14.0) | 112  (43.4) | | Yes | NA | | | | | | | | | | | | | | | 19  (5,9,5) |
| Jonge  (6) | 2009 | White  -Netherlands | C | 1 | 93 (38.0) | | 22 (9.0) | | 130 (53.1) | | 223 (45.0) | 54 (10.9) | 219 (44.2) | | Yes | 110  (44.9) | 35  (14.3) | | 100  (40.8) | | | 229  (47.0) | | 45  (9.2) | | 213  (43.7) | | | Yes | | 16  (3,8,5) |
| Winkel  (7) | 2011 | White  -Netherlands | C | 1 | 35 (42.2) | | 5  (6.0) | | 43 (51.8) | | 73 (49.7) | 10 (6.8) | 64 (43.5) | | Yes | 41  (50.0) | 10  (12.2) | | 31  (37.8) | | | 63  (42.6) | | 16  (10.8) | | 69  (46.6) | | | Yes | | 19  (6,8,5) |
| Schnakenberg (8) | 2005 | White  -Germany | C | 1 | 201  (45.4) | | 47  (10.6) | | 195  (44.0) | | 152  (40.1) | 43  (11.4) | 184  (48.6) | | Yes | 194  (43.8) | 45  (10.2) | | 204  (46.1) | | | 153  (40.4) | | 52  (13.7) | | 174  (45.9) | | | Yes | | 18  (5,8,5) |
| Thirumaran (9) | 2005 | White  -Germany | C | 1 | 195 (43.1) | | 59 (13.0) | | 199 (43.9) | | 681 (47.0) | 167 (11.5) | 600 (41.4) | | Yes | 198  (44.5) | 52  (11.7) | | 195  (43.8) | | | 660  (45.4) | | 149  (10.3) | | 644  (44.3) | | | Yes | | 15  (4,6,5) |
| Oliveira  (10) | 2005 | Mixed  - Portugal | C | 1 | 50  (48.5) | | 5  (4.9) | | 48 (46.6) | | 57 (51.4) | 9  (8.1) | 45 (40.5) | | Yes | 36  (35.0) | 9  (8.7) | | 58  (56.3) | | | 54  (48.7) | | 9  (8.1) | | 48  (43.2) | | | Yes | | 16  (5,7,4) |
| Skibola  (11) | 1999 | White  -UK | A | 1 | 29  (42.0) | | 5  (7.3) | | 35  (50.7) | | 39  (34.2) | 14  (12.3) | 61  (53.5) | | Yes | 45  (65.2) | 1  (1.5) | | 23  (33.3) | | | 49  (43.0) | | 11  (9.7) | | 54  (47.4) | | | Yes | | 18  (5,8,5) |
| Wiemels  (12) | 2001 | White  -UK | C | 5 | 91 (42.1) | | 27  (12.5) | | 98  (45.3) | | 79 (39.5) | 32 (16.0) | 89 (44.5) | | No | 109  (51.9) | 11  (5.2) | | 90  (42.9) | | | 93  (46.7) | | 23  (11.6) | | 83  (41.7) | | | Yes | | 18  (5,7,6) |
| Rudd  (13) | 2004 | White  -UK | A | 4 | 381  (45.8) | | 90  (10.8) | | 361  (43.4) | | 397  (44.8) | 106  (12.0) | 383  (43.2) | | Yes | 397  (47.7) | | 72  (8.7) | | 363  (43.6) | | | 412  (46.5) | | 85  (9.6) | | 389  (43.9) | | | Yes | 19  (6,9,4) |
| Lightfoot  (14) | 2010 | White  -UK | C | 5 | 362 (41.0) | | 101  (11.4) | | 421  (47.6) | | 317  (41.7) | 84 (11.1) | 359 (47.2) | | Yes | 408  (52.3) | | 73  (9.3) | | 305  (38.8) | | | 350  (46.1) | | 77  (10.1) | | 332  (43.7) | | | Yes | 15  (3,7,5) |
| Chiusolo (15) | 2004 | White  -Italy | A | 1 | 71  (40.8) | | 38  (21.8) | | 65  (37.4) | | 55  (50.0) | 20  (18.2) | 35  (31.8) | | Yes | 47  (41.2) | | 12  (10.5) | | 55  (48.3) | | | 126  (49.0) | | 21  (8.2) | | 110  (42.8) | | | Yes | 20  (6,8,6) |
| Gemmati (16) | 2004 | White  -Italy | A | 1 | 53  (46.5) | | 9  (7.9) | | 52  (45.6) | | 128  (49.8) | 51  (19.8) | 78  (30.4) | | Yes | 92  (52.9) | | 9  (5.2) | | 73  (42.0) | | | 56  (50.9) | | 5  (4.6) | | 49  (44.6) | | | Yes | 20  (7,7,6) |
| **America** | | | | | | | | | | | | | | | | | | | | | | | | | | | | | | | |
| Krajinovic (17) | 2004 | Mixed  -Canada | C | 1 | 127  (47.0) | | 31  (11.5) | | 112  (41.5) | | 128  (42.7) | 46  (15.3) | 126  (42.0) | | Yes | 151  (55.9) | | 12  (4.4) | | 107  (39.6) | | | 150  (50.0) | | 31  (10.3) | | 119  (39.7) | | | Yes | 15  (4,5,6) |

Table S1 Cont.

| First Author  (Reference number) | Year | Ethnicity  -Country | Group | MTHFR 677 | | | | | | | | MTHFR 1298 | | | | | | | Quality Score | | |
| --- | --- | --- | --- | --- | --- | --- | --- | --- | --- | --- | --- | --- | --- | --- | --- | --- | --- | --- | --- | --- | --- |
|  |  |  |  | Cases, Cancer Site (CS), n (%) | | | | Controls, n (%) | | | | Cases n (%) | | | Controls n (%) | | | |  |  | |
|  |  |  |  | LT | CT | TT | CC | CT | TT | CC | HWE | AA | CC | AC | AA | CC | AC | HWE |  | |  |

| Franco et  (18) | 2001 | Mixed  -Brazil | C | 1 | 28  (40.0) | | 6  (8.6) | 36  (51.4) | 36  (50.7) | 13  (18.3) | 22  (31.0) | Yes | 36  (50.7) | 5  (7.0) | 30  (42.2) | 41  (57.8) | 2  (2.8) | 28  (39.4) | Yes | 15  (4,5,6) |
| --- | --- | --- | --- | --- | --- | --- | --- | --- | --- | --- | --- | --- | --- | --- | --- | --- | --- | --- | --- | --- |
| Zanrosso  (19) | 2006 | White  -Brazil | C | 1 | 35  (40.7) | | 8  (9.3) | 43  (50.0) | 50  (42.0) | 10  (8.4) | 59  (49.6) | Yes | 48  (53.3) | 7  (7.8) | 35  (38.9) | 62  (51.7) | 8  (6.7) | 50  (41.7) | Yes | 19  (6,7,6) |
| Zanrosso  (19) | 2006 | Mixed  -Brazil | C | 1 | 21  (26.6) | | 5  (6.3) | 53  (67.1) | 32  (40.5) | 10  (12.7) | 37  (46.8) | Yes | 35  (44.9) | 4  (5.1) | 39  (50.0) | 49  (62.0) | 4  (5.1) | 26  (32.9) | Yes | 19  (6,7,6) |
| Barbosa  (20) | 2008 | White  -Brazil | A | 2 | 8  (29.6) | | 2  (7.4) | 17  (63.0) | 29  (29.0) | 6  (6.0) | 65  (65.0) | Yes | 15  (55.6) | 1  (3.7) | 11  (40.7) | 63  (63.0) | 5  (5.0) | 32  (32.0) | Yes | 17  (5,6,6) |
| Barbosa  (20) | 2008 | Mixed  -Brazil | A | 4 | 19  (28.4) | | 2  (3.0) | 46  (68.7) | 29  (29.0) | 6  (6.0) | 65  (65.0) | Yes | 41  (61.2) | 3  (4.5) | 23  (34.3) | 63  (63.0) | 5  (5.0) | 32  (32.0) | Yes | 17  (4,7,6) |
| Lordelo  (21) | 2012 | White  -Brazil | A | 4 | 21  (51.2) | | 5  (12.2) | 15  (36.6) | 66  (42.6) | 15  (9.68) | 74  (47.7) | Yes | 26  (63.4) | 0  (0.0) | 15  (36.6) | 68  (43.9) | 8  (5.2) | 79  (51.0) | Yes | 17  (4,7,6) |
| Lordelo  (21) | 2012 | Mixed  -Brazil | A | 4 | 26  (40.6) | | 7  (10.9) | 31  (48.4) | 48  (40.7) | 4  (3.4) | 66  (55.9) | Yes | 35  (54.7) | 1  (1.6) | 28  (43.8) | 51  (43.2) | 3  (2.5) | 64  (54.2) | Yes | 17  (4,7,6) |
| Siliva  (22) | 2013 | Mixed  -Brazil | C | 5 | 53  (36.8) | | 9  (6.2) | 82  (56.9) | 108  (48.2) | 21  (9.4) | 95  (42.4) | Yes | 13  (41.9) | 5  (16.1) | 13  (41.9) | 147  (59.3) | 19  (7.7) | 82  (33.1) | Yes | 19  (5,9,5) |
| **East Asia** | | | | | | | | | | | | | | | | | | | | |
| Jiang  (23) | 2004 | Asian  -China | C | 1 | | 14  (48.3) | 0  (0.0) | 15  (51.7) | 41  (61.2) | 8  (11.9) | 18  (26.9) | No | NA | | | | | | | 15  (5,6,4) |
| Yu  (24) | 2006 | Asian  -China | C | 1 | | 14  (27.5) | 7  (13.7) | 30  (58.8) | 23  (43.4) | 10  (18.9) | 20  (37.7) | Yes | NA | | | | | | | 15  (5,6,4) |
| Zhang  (25) | 2007 | Asian  -China | A | 1 | | 17  (37.0) | 4  (8.7) | 25  (54.4) | 41  (51.3) | 13  (16.3) | 26  (32.5) | Yes | 31  (67.4) | 1  (2.2) | 14  (30.4) | 51  (63.8) | 4  (5.0) | 25  (31.3) | Yes |  |
| Liu  (26) | 2008 | Asian  -China | C | 1 | | 23  (27.7) | 26  (31.3) | 34  (41.0) | 36  (43.4) | 9  (10.8) | 38  (45.8) | Yes | NA | | | | | | | 20  (5,9,6) |
| Tong  (27) | 2010 | Asian  -China | C | 1 Yes | | 192  (53.2) | 34  (9.4) | 135  (37.4) | 257  (51.1) | 73  (14.5) | 173  (34.4) | Yes | 256  (70.9) | 15  (4.2) | 90  (24.9) | 342  (67.3) | 24  (4.7) | 142  (28.0) | Yes | 18  (4,8,6) |
| Lv  (28) | 2010 | Asian  -China | A | 1 | | 65  (51.2) | 24  (18.9) | 38  (30.0) | 83  (45.6) | 27  (14.8) | 72  (39.6) | Yes | 86  (67.7) | 5  (3.9) | 36  (28.4) | 111  (61.0) | 4  (2.2) | 67  (36.8) | Yes | 20  (5,9,6) |
| Lv  (28) | 2011 | Asian  -China | C | 1 | | 70  (39.8) | 44  (25.0) | 62  (35.2) | 87  (51.2) | 34  (20.0) | 49  (28.8) | Yes | 121  (68.8) | 5  (2.8) | 50  (28.4) | 121  (71.2) | 2  (1.2) | 47  (31.3) | Yes | 20  (5,9,6) |
| Yang  (29) | 2011 | Asian  -China | C | 1 | | 101  (43.7) | 76  (32.9) | 54  (22.9) | 168  (45.8) | 115  (31.3) | 84  (22.9) | Yes | 167  (72.3) | 3  (1.3) | 60  (26.4) | 254  (69.2) | 7  (1.9) | 106  (28.9) | Yes | 17  (5,6,6) |
| Yang  (29) | 2011 | Asian  -China | A | 1 | | 79  (60.8) | 20  (15.4) | 31  (22.9) | 168  (45.8) | 115  (31.3) | 84  (22.9) | Yes | 85  (65.4) | 4  (3.1) | 41  (31.5) | 254  (69.2) | 7  (1.9) | 106  (28.9) | Yes | 17  (5,6,6) |
| Zheng  (30) | 2013 | Asian  -China | C | 5 | | 28  (32.2) | 16  (18.4) | 43  (49.4) | 54  (45.0) | 10  (8.3) | 56  (46.7) | Yes | 60  (69.0) | 6  (6.9) | 21  (24.1) | 91  (75.8) | 8  (6.8) | 21  (17.5) | Yes | 17  (4,7,6) |
| Table S1. Cont. | | | | | | | | | | | | | | | | | | | | |
| First Author  (Reference number) | Year | Ethnicity  -Country | Group | MTHFR 677 | | | | | | | | | MTHFR 1298 | | | | | | | Quality Score |
|  |  |  |  | Cases, Cancer Site (CS), n (%) | | | | | Controls, n (%) | | | | Cases n (%) | | | Controls n (%) | | | |  |
|  |  |  |  | LT | CT | | TT | CC | CT | TT | CC | HWE | AA | CC | AC | AA | CC | AC | HWE |  |
| Li  (31) | 2014 | Asian  -China | C | 1 | 44  (44.9) | | 26  (26.5) | 28  (28.6) | 49  (52.7) | 15  (16.1) | 29  (31.2) | Yes | 54  (55.1) | 2  (2.0) | 42  (42.9) | 67  (72.0) | 1  (1.1) | 25  (26.9) | Yes | 19  (5,9,5) |
| Hur  (32) | 2006 | Asian  -Korea | C | 1 | 44  (49.4) | | 15  (16.9) | 30  (33.7) | 80  (40.0) | 40  (20.0) | 80  (40.0) | Yes | 64  (77.9) | 2  (2.3) | 23  (25.8) | 116  (58.0) | 6  (3.0) | 78  (39.0) | Yes | 18  (5,7,6) |
| Hur  (32) | 2006 | Asian  -Korea | A | 2 | 30  (54.6) | | 7  (12.7) | 18  (32.7) | 80  (40.0) | 40  (20.0) | 80  (40.0) | Yes | 33  (60.0) | 3  (5.5) | 19  (34.6) | 116  (58.0) | 6  (3.0) | 78  (39.0) | Yes | 18  (5,7,6) |
| Hur  (32) | 2006 | Asian  -Korea | A | 4 | 17  (42.5) | | 10  (25.0) | 13  (32.5) | 80  (40.0) | 40  (20.0) | 80  (40.0) | Yes | 31  (77.5) | 2  (5.0) | 7  (17.5) | 116  (58.0) | 6  (3.0) | 78  (39.0) | yes | 18  (5,7,6) |
| Kim  (33) | 2006 | Asian  -Korea | C | 1 | 38  (57.6) | | 11  (16.7) | 17  (25.8) | 55  (55.0) | 21  (21.0) | 24  (24.0) | Yes | 38  (61.3) | 1  (1.6) | 23  (31.7) | 77  (77.0) | 2  (2.0) | 21  (21.0) | Yes | 16  (6,4,6) |
| Kim  (34) | 2009 | Asian  -Korea | A | 6 | 51  (47.7) | | 27  (25.2) | 29  (21.1) | 863  (50.8) | 297  (17.5) | 540  (31.8) | Yes | 439  (66.9) | 18  (2.7) | 199  (30.3) | 1147  (67.5) | 53  (3.1) | 500  (29.4) | Yes | 19  (6,7,6) |
| Moon  (35) | 2006 | Asian  -Korea | A | 6 | 152  (48.3) | | 59  (18.7) | 104  (33.0) | 196  (45.2) | 94  (21.7) | 144  (33.2) | Yes | 212  (67.3) | 15  (4.8) | 88  (27.9) | 307  (70.7) | 7  (1.6) | 120  (27.7) | Yes | 17  (6,5,6) |
| Oh  (36) | 2007 | Asian  -Korea | A | 1 | 55  (46.6) | | 14  (11.9) | 49  (41.5) | 229  (53.6) | 60  (14.1) | 138  (32.3) | No | 72  (67.3) | 2  (1.9) | 33  (30.8) | 293  (68.6) | 8  (1.9) | 126  (29.5) | Yes | 18  (6,7,5) |
| Liu  (37) | 2008 | Asian  -Taiwan | C | 1 | 27  (28.7) | | 12  (12.8) | 55  (58.5) | 56  (32.6) | 15  (8.7) | 101  (58.7) | Yes | 57  (63.3) | 3  (3.3) | 30  (33.3) | 103  (60.6) | 11  (6.5) | 56  (32.9) | Yes | 20  (5,9,6) |

| **South Asia** |
| --- |

| Yeoh  (38) | 2009 | | Asian  -Singapore | | C | 1 | 111  (34.9) | | 23  (7.2) | | 184  (57.9) | | | 150  (43.5) | 32  (9.3) | | 163  (47.3) | | Yes | | 259  (49.2) | | 49  (9.3) | | 218  (41.4) | | 368  (55.2) | | 54  (8.1) | 245  (36.7) | | | | Yes | | | | 22  (7,9,6) | | |  | |
| --- | --- | --- | --- | --- | --- | --- | --- | --- | --- | --- | --- | --- | --- | --- | --- | --- | --- | --- | --- | --- | --- | --- | --- | --- | --- | --- | --- | --- | --- | --- | --- | --- | --- | --- | --- | --- | --- | --- | --- | --- | --- | --- |
| Alcasabas  (39) | 2008 | | Asian  -Philippines | | C | 1 | 41  (21.7) | | 3  (1.6) | | 145  (76.7) | | | 66  (16.8) | 6  (1.5) | | 322  (81.7) | | Yes | | 56  (29.8) | | 38  (20.2) | | 94  (50.0) | | 160  (40.6) | | 56  (14.2) | 178  (45.2) | | | | Yes | | | | 22  (8,8,6) | | |  | |
| Giovannetti (40) | 2008 | | Asian  -Indonesia | | C | 1 | 11  (16.9) | | 3  (4.6) | | 51  (78.5) | | | 6  (18.8) | 0  (0.0) | | 26  (81.3) | |  | | NA | | | | | | | | | | | | | | | | | | 16  (5,5,6) | | |  |
| Giovannetti (40) | 2008 | | Caucasian  -Indonesia | | C | 1 | 234  (46.5) | | 45  (9.0) | | 224  (44.5) | | | 31  (36.1) | 8  (9.3) | | 47  (54.7) | | Yes | | NA | | | | | | | | | | | | | | | | | | 16  (5,5,6) | | |  |
| Chan  (41) | 2011 | | Asian  -Indonesia | | C | 1 | 43  (23.3) | | 2  (1.1) | | 140  (75.7) | | | 51  (28.8) | 4  (2.3) | | 122  (68.9) | | Yes | | 99  (55.0) | | | 8  (4.4) | | 73  (40.6) | 82  (49.4) | 9  (5.4) | | | 75  (45.2) | | | | Yes | | | | 8  (4.4) | | |  |
| Reddy  (42) | 2006 | | Asian  -India | | C | 1 | 77  (57.0) | | 7  (5.2) | | 51  (37.8) | | | 58  (40.9) | 5  (3.5) | | 79  (55.6) | | Yes | | 41  (30.4) | | 11  (8.2 | | 83  (61.5) | | 65  (45.8) | | 9  (6.3) | | | 68  (47.9) | | | | Yes | | 20  (6,8,6) | |  | | |
| Sadananda  (54) | | 2010 | | Asian  -India | C | 1 | | 13  (15.1) | | 0  (0.0) | | 73  (84.9) | 14  (14.1) | | | 0  (0.0) | | 85  (85.9) | | Yes | 20  (23.3) | 15  (17.4) | | | 51  (59.3) | | 28  (28.3) | | 16  (16.2) | | | | 55  (55.6) | | | | Yes | 18  (6,6,6) | | | | |
| Sood  (43) | | 2010 | | Asian  -India | C | 1 | | 38  (40.0) | | 3  (3.2) | | 54  (56.8) | 71  (27.8) | | | 11  (4.3) | | 173  (67.8) | | Yes | 30  (31.6) | 19  (20.0) | | | 46  (48.4) | | 59  (23.1) | | 50  (19.6) | | | | 146  (57.3) | | | | No | 17  (5,7,5) | | | | |
| Hussain  (44) | | 2012 | | Asian  -India | A | 8 | | 140  (33.6) | | 39  (9.4) | | 238  (57.1) | 180  (36.6) | | | 61  (12.4) | | 251  (51.0) | | No | NA | | | | | | | | | | | | | | | | | | 20  (6,9,5) |  | | |
| Table S1. Cont. | | | | | | | | | | | | | | | | | | | | | | | | | | | | | | | | | | | | | | | | | | |

| **Middle East** |
| --- |

| Ismail  (45) | 2009 | Asian  -Jordan | A | 4 | 67  (45.0) | 19  (12.8) | 63  (42.3) | 66  (38.8) | 10  (5.9) | 94  (55.3) | Yes | 59  (39.6) | 22  (14.8) | 68  (45.6) | 76  (44.7) | 13  (7.7) | 81  (47.7) | Yes | 17  (5,6,6) |
| --- | --- | --- | --- | --- | --- | --- | --- | --- | --- | --- | --- | --- | --- | --- | --- | --- | --- | --- | --- |
| Azhar  (46) | 2012 | Asian  -Iran | C | 1 | 31 (43.1) | 6  (8.3) | 35 (48.6) | 34 (31.2) | 10 (9.2) | 65 (59.6) | Yes | 26  (36.1) | 16  (22.2) | 30  (41.7) | 43  (39.5) | 17  (15.6) | 49  (45.0) | Yes | 16  (5,5,6) |
| Vahid  (47) | 2010 | Asian  -Iran | A | 6 | 50  (34.7) | 12  (8.3) | 82  (56.9) | 37  (38.2) | 4  (4.1) | 56  (57.7) | Yes | 52  (36.1) | 24  (16.7) | 68  (47.2) | 39  (40.2) | 22  (22.7) | 36  (37.1) | Yes | 17  (5,8,4) |
| Balta  (48) | 2003 | Mixed  -Turkey | C | 1 | 60 (42.3) | 11  (7.8) | 71 (50.0) | 87 (47.0) | 8  (4.3) | 90 (48.7) | No | 70  (49.7) | 11  (7.8) | 60  (42.6) | 90  (48.7) | 8  (4.3) | 87  (47.0) | No | 20  (7,7,6) |
| Deligezer  (49) | 2003 | Mixed  -Turkey | A | 7 | 107  (44.0) | 14  (5.8) | 122  (50.2) | 73  (45.3) | 14  (8.7) | 74  (46.0) | Yes | NA | | | | | | | 15  (3,6,6) |
| Timuragaoglu  (50) | 2006 | White  -Turkey | A | 1 | 12  (36.4) | 9  (27.3) | 12  (36.4) | 36  (43.9) | 10  (12.2) | 36  (43.9) |  | NA | | | | | | |  |
| Kantar.  (51) | 2009 | Mixed  -Turkey | C | 1 | 9 (45.0) | 3 (15.0) | 8 (40.0) | 5  (29.4) | 1  (5.9) | 11 (64.7) | Yes | 6  (30.0) | 2  (10.0) | 12  (12.0) | 5  (29.4) | 5  (29.4) | 7  (41.2) | Yes | 16  (2,8,6) |
| **Africa** | | | | | | | | | | | | | | | | | | | |
| Kamel  (52) | 2007 | Asian  -Egypt | C | 1 | 42  (47.7) | 7  (8.0) | 39  (44.3) | 135  (43.4) | 20  (6.4) | 156  (50.2) | Yes | 58  (65.9) | 8  (9.1) | 22  (25.0) | 141  (45.5) | 29  (9.4) | 140  (45.2) | Yes | 18  (4,9,5) |
| Khorshied  (53) | 2014 | Mixed  -Egypt | A | 4 | 45  (46.4) | 11  (11.3) | 41  (42.3) | 52  (40.0) | 13  (10.0) | 65  (50.0) | Yes | 54  (55.7) | 6  (6.2) | 37  (38.1) | 55  (42.3) | 10  (7.7) | 65  (40.0) | Yes | 19  (5,9,) |

Notes:

Group: A = adult, C = child (Age under 20 years old); HWE = Hardy Weinberg Equilibrium;

LT = leukemia type 1=ALL (acute lymphoblastic leukemia), 2 = AML (acute myeloid leukemia), 3 = CLL (chronic lymphoblastic leukemia), 4 = CML (chronic myeloid leukemia), 5 = ALL+AML, 6 = AML+CML, 7 = ALL+AML+CML, 8 = ALL+AML+CLL+CML.

Quality score range = 0-30: external validity = 0-11, Internal Validity = 0-12, report quality = 0-7.

NA = not available; ^a^10 European countries: Denmark, France, Greece, Germany, Italy, Netherlands, Norway, Spain, Sweden, United Kingdom (UK).
