# Supplementary material for: Meta-prediction of MTHFR gene polymorphism-mutations, air pollution, and risks of leukemia among world populations
Source: Oncotarget. 2016 Dec 10;8(3):4387–98. doi: 10.18632/oncotarget.13876 (PMC5354840; doi:10.18632/oncotarget.13876)
Supplement: Supplementary file 3 [file oncotarget-08-4387-s003.docx]

Table S2. Pooled analysis: *MTHFR 677* genotypes and risks of leukemia by continents (62 study groups).

| **Genotype by Race or Ethnicity (number of studies)** | **Leukemia Case**  **(N = 10033)**  **n (%)** | **Control**  **(N = 15835)**  **n (%)** | **Test of Heterogeneity** | | | **Statistical Model** | **Test of Association** | |
| --- | --- | --- | --- | --- | --- | --- | --- | --- |
|  |  |  | Q | *p* | I^2^ |  | Risk Ratio  (95% Cl) | *p* |
| **TT (62)** | 1155 (11.51) | 2091 (13.20) | 97.47 | 0.0021 | 37.4% | Random | 0.95 (0.85 to 0.99) | 0.3410 |
| Europe (16) | 431 (11.04) | 707 (12.25) | 17.25 | 0.3692 | 7.3% | Fixed | 0.89 (0.79 to 0.99) | **0.0400** |
| East Asia (19) | 432 (18.76) | 1036 (18.77) | 47.61 | 0.0002 | 62.2% | Random | 1.02 (0.84 to 1.24) | 0.8114 |
| South Asia (9) | 125 (6.27) | 127 (6.28) | 3.111 | 0.8745 | 0% | Fixed | 0.83 (0.64 to 1.07) | 0.1551 |
| America (9) | 75 (8.84) | 131 (10.35) | 9.775 | 0.2812 | 18.2% | Fixed | 0.80 (0.61 to 1.05) | 0.1061 |
| Middle East (7) | 74 (9.21) | 57 (6.94) | 8.253 | 0.1428 | 39.4% | Fixed | 1.39 (0.99 to 1.97) | 0.0605 |
| Africa (2) | 18 (9.73) | 33 (7.48) | 0.023 | 0.8795 | 0% | Fixed | 1.18 (0.67 to 2.06) | 0.5626 |
| **CT (62)** | 4212 (41.98) | 6929 (43.75) | 109.35 | 0.0002 | 43.3% | Random | 0.97 (0.94 to 1.02) | 0.3535 |
| Europe (16) | 1682 (43.11) | 2605 (45.17) | 22.04 | 0.1418 | 27.4% | Fixed | 0.96 (0.91 to 1.00) | 0.0508 |
| East Asia (19) | 1061 (46.07) | 2646 (47.95) | 40.97 | 0.0015 | 56.1% | Random | 0.97 (0.89 to 1.06) | 0.5017 |
| South Asia (9) | 708 (35.52) | 627 (31.01) | 24.05 | 0.0022 | 66.7% | Random | 1.08 (0.90 to 1.29) | 0.3894 |
| America (9) | 338 (39.86) | 526 (41.55) | 10.87 | 0.2089 | 26.4 | Fixed | 0.95 (0.85 to 1.05) | 0.3080 |
| Middle East (7) | 336 (41.84) | 338 (41.17) | 5.937 | 0.3124 | 15.8% | Fixed | 1.03 (0.91 to 1.17) | 0.6116 |
| Africa (2) | 87 (47.03) | 187 (42.40) | 0.071 | 0.7896 | 0% | Fixed | 1.13 (0.93 to 1.37) | 0.2319 |
| **CC (62)** | 4666 (46.51) | 6815 (43.04) | 134.38 | <0.0001 | 53.9% | Random | 1.04 (1.00 to 1.09) | 0.0812 |
| Europe (16) | 1788 (45.83) | 2455 (42.57) | 33.87 | 0.0057 | 52.8% | Random | 1.12 ( 1.04 to 1.21) | **0.0021** |
| East Asia (19) | 810 (35.17) | 1836 (33.27) | 28.45 | 0.0555 | 36.7% | Fixed | 1.03 (0.96 to 1.11) | 0.3725 |
| South Asia (9) | 1160 (58.20) | 1268 (62.71) | 29.92 | 0.0002 | 73.3% | Random | 0.97 (0.88 to 1.07) | 0.5749 |
| America (9) | 435 (51.30) | 609 (48.10) | 18.06 | 0.0208 | 55.7% | Random | 1.09 (0.95 to 1.26) | 0.2058 |
| Middle East (7) | 393 (48.94) | 426 (51.89) | 8.346 | 0.1382 | 40.1% | Fixed | 0.91 (0.82 to 1.03) | 0.1300 |
| Africa (2) | 80 (43.24) | 221 (50.11) | 0.050 | 0.8236 | 0% | Fixed | 0.87 (0.71 to 1.05) | 0.1455 |
| **TT+CT (62)** | 5367 (53.49) | 9020 (56.96) | 121.35 | <0.0001 | 49.7% | Random | 0.98 (0.94 to 1.01) | 0.1916 |
| Europe (16) | 1403 (52.74) | 2462 (56.64) | 25.56 | 0.0429 | 41.3% | Random | 0.93 (0.88 to 0.98) | **0.0098** |
| **CC+CT (62)** | 8878 (88.48) | 13744 (86.79) | 111.99 | <0.0001 | 46.4 | Random | 1.01 (1.00 to 1.02) | 0.1114 |
| **C allele** | 6772 (67.48) | 10280 (64.92) | 74.22 | 0.1191 | 17.8% | Fixed | 1.02 (1.00 to 1.04) | **0.0072** |
| **T allele** | 3261 (32.50) | 5556 (35.08) | 69.10 | 0.2227 | 11.7% | Fixed | 0.97 (0.93 to 1.00) | **0.0074** |
| Subgroups |  |  |  |  |  |  |  |  |
| *TT Risk>1* | 617 | 1177 |  |  |  |  |  |  |
| TT (5) | 52 (8.43) | 64 (5.44) | 1.92 | 0.7503 | 0% | Fixed | 1.45 (1.01 to 2.08) | **0.0452** |
| CT (5) | 222 (35.98) | 375 (31.86) | 2.28 | 0.6842 | 0% | Fixed | 1.12 (0.98 to 1.28) | 0.0878 |
| CC (5) | 343 (55.59) | 738 (62.70) | 3.88 | 0.4224 | 0% | Fixed | 0.90 (0.83 to 0.97) | **0.0080** |
| TT+CT (5) | 274 (44.41) | 439 (37.30) | 2.25 | 0.6944 | 0% | Fixed | 1.17 (1.05 to 1.31) | **0.0062** |
| CC+CT (5) | 565 (91.57) | 1113 (94.56) | 7.58 | 0.1083 | 47.2% | Fixed | 0.97 (0.95 to 1.00) | 0.0522 |
| *TT Risk<1* | 1217 | 2122 |  |  |  |  |  |  |
| TT (8) | 101 (8.30) | 253 (11.92) | 2.31 | 0.9404 | 0% | Fixed | 0.71 (0.56 to 0.90) | **0.0004** |
| CT (8) | 506 (41.65) | 964(45.43) | 10.2 | 0.1797 | 31.1 | Fixed | 0.91 (0.84 to 0.99) | **0.0252** |
| CC (8) | 610 (50.12) | 905 (42.65) | 8.31 | 0.3064 | 15.7% | Fixed | 1.17 (1.08 to 1.27) | **0.0001** |
| TT+CT (8) | 607 (49.88) | 1217 (57.35) | 9.29 | 0.2326 | 24.6% | Fixed | 0.87 (0.81 to 0.93) | **<0.0001** |
| CC+CT (8) | 1116 (91.70) | 1869 (88.08) | 4.30 | 0.7451 | 0% | Fixed | 1.04 (1.01 to 1.06) | **0.0022** |
| *TT Risk~1* | 8199 | 12536 |  |  |  |  |  |  |
| TT (49) | 1020 (12.17) | 1774 (14.15) | 82.26 | 0.0011 | 42.9% | Random | 0.97 (0.86 to 1.09) | 0.6112 |
| CT (49) | 3484 (42.49) | 5590 (44.59) | 87.45 | 0.0004 | 45.1% | Random | 0.98 (0.93 to 1.04) | 0.5400 |
| CC (49) | 3713 (45.29) | 5172 (41.26) | 95.74 | <0.0001 | 49.9% | Random | 1.03 (0.98 to 1.08) | 0.2285 |
| TT+CT (49) | 4486 (54.71) | 7364 (58.74) | 90.87 | 0.0002 | 47.2% | Random | 0.98 (0.94 to 1.02) | 0.3353 |
| CC+CT (49) | 7197 (87.78) | 10762 (85.85) | 94.18 | <0.0001 | 50.1% | Random | 1.01 (0.99 to 1.03) | 0.2626 |

*Note:*

*MTHFR =* methylenetetrahydrofolate reductase; CI = confidence interval; TT = *MTHFR* homozygous genotype TT; CC = *MTHFR* homozygous genotype CC; CT = *MTHFR* homozygous genotype CT; TT+CT = *MTHFR* homozygous genotype TT plus CT; CC+CT = *MTHFR* homozygous genotype CC plus CT.

*Europe: Greece, Germany, Italy, Netherlands, Serbia, Slovenia, United Kingdom, Portugal; America: Canada, Brazil, Middle East: Iran, Jordan, Turkey; East Asia: China, South Korea and Taiwan; South Asia: Singapore, Philippines, Indonesia and India; Africa: Egypt.*

*TT Risk>1countries included: Philippines, Taiwan, Jordan and Egypt.*

*TT Risk<1countries included: Canada, Netherlands, Portugal, Serbia, Singapore and Slovenia.*

*TT Risk~1countries included: Brazil, China, Germany, Greece, India, Indonesia, Iran, Italy, South Korean, Turkey and United Kingdom.*
