# Supplementary material for: Meta-prediction of MTHFR gene polymorphism-mutations, air pollution, and risks of leukemia among world populations
Source: Oncotarget. 2016 Dec 10;8(3):4387–98. doi: 10.18632/oncotarget.13876 (PMC5354840; doi:10.18632/oncotarget.13876)
Supplement: Supplementary file 4 [file oncotarget-08-4387-s004.docx]

Table S3. Pooled analysis: *MTHFR* 1298 genotypes and risk of leukemia by race (50 Study groups).

| **Genotype**  **(number of studies)** | **Leukemia Case**  **n (%)** | **Control**  **n (%)** | **Test of Heterogeneity** | | | **Statistical Model** | **Test of Association** | |
| --- | --- | --- | --- | --- | --- | --- | --- | --- |
|  |  |  | Q | *p* | I^2^ |  | Risk Ratio (95% Cl) | *p* |
| **CC (50)** | 687 (7.75) | 1036 (7.24) | 56.19 | 0.2235 | 12.8% | Fixed | 1.02 (0.93 to 1.12) | 0.6587 |
| European (12) | 333 (9.41) | 497 (10.03) | 21.80 | 0.0398 | 45% | Random | 1.02 (0.84 to 1.24) | 0.9592 |
| East Asian (16) | 87 (3.26) | 156 (2.93) | 11.71 | 0.7007 | 0% | Fixed | 1.09 (0.84 to 1.43) | 0.5019 |
| South Asian (6) | 140 (11.57) | 194 (11.26) | 2.05 | 0.8424 | 0% | Fixed | 1.17 (0.95 to 1.44) | 0.1248 |
| American (9) | 38 (5.14) | 85 (6.58) | 11.46 | 0.1768 | 30.2% | Random | 0.93 (0.56 to 1.56) | 0.7910 |
| Middle East (5) | 75 (14.26) | 65 (11.25) | 9.29 | 0.0542 | 57% | Fixed | 1.18 (0.87 to 1.61) | 0.2624 |
| African (2) | 14 (7.57) | 39 (8.86) | 0.335 | 0.5626 | 0% | Fixed | 0.90 (0.50 to 1.63) | 0.7405 |
| **AC (50)** | 3454(38.95) | 5527 (38.65) | 79.48 | 0.005 | 37.1% | Random | 0.99 (0.95 to 1.04) | 0.6651 |
| European (12) | 1514 (42.79) | 2187 (44.17) | 13.28 | 0.3485 | 9.7% | Fixed | 0. 97 (0.92 to 1.02) | 0.1791 |
| East Asian (16) | 777 (29.10) | 1596 (30.01) | 25.51 | 0.0436 | 41.2% | Random | 0.99 (0.89 to 1.10) | 0.867 |
| South Asian (6) | 565(46.69) | 767 (44.52) | 9.28 | 0.0545 | 56.9% | Random | 1.05 (0.92 to 1.20) | 0.4436 |
| American (9) | 301 (40.73) | 512 (39.66) | 11.35 | 0.1824 | 29.5% | Fixed | 1.00 (0.94 to 1.08) | 0.7985 |
| Middle East (5) | 238(42.25) | 260 (44.98) | 20.78 | 0.0001 | 85.6% | Random | 1.02 (0.67 to 1.57) | 0.9131 |
| African (2) | 59 (31.89) | 205 (46.59) | 1.711 | 0.1908 | 41.6% | Fixed | 0.65 (0.51 to 0.83) | 0.0006 |
| **AA (50)** | 4727 (53.30) | 7738 (54.11) | 113.2 | <0 .0001 | 55.9% | Random | 1.01 (0.97 to 1.05) | 0.6021 |
| European (12) | 1691 (47.79) | 2267 (45.89) | 22.27 | 0.0346 | 46.1% | Random | 1.03 (0.96 to 1.10) | 0.3962 |
| East Asian (16) | 1806 (67.64) | 3566 (67.06) | 30.18 | 0.0113 | 50.3% | Random | 1.00 (0.95 to 1.05) | 0.8092 |
| South Asian (6) | 505 (41.74) | 762 (44.23) | 15.36 | 0.004 | 74% | Random | 0.91 (0.74 to 1.19) | 0.3592 |
| American (9) | 400 (54.13) | 694 (53.76) | 16.34 | 0.0378 | 51% | Random | 0.93 (0.66 to 1.30) | 0.6698 |
| Middle East (5) | 213 (40.49) | 253 (43.77) | 4.23 | 0.2376 | 29.1% | Fixed | 1.03 (0.88 to 1.12) | 0.7097 |
| African (2) | 112 (60.54) | 196 (44.55) | 0.335 | 0.5626 | 0% | Fixed | 1.39 (1.19 to 1.63) | <0.0001 |
| **CC+AC (50)** | 4141 (46.70) | 6563 (45.89) | 103.5 | <0.0001 | 52.6% | Random | 1.00 (0.95 to 1.05) | 0.9880 |
| African (2) | 73 (39.46) | 244 (55.45) | 1.008 | 0.3152 | 0% | Fixed | 0.69 (0.56 to 0.85) | 0.0004 |
| **AA+AC (50)** | 8181 (92.25) | 13265 (92.76) | 62.8 | 0.0883 | 22% | Random | 1.00 (0.99 to 1.01) | 0.9706 |
| **A Allele** | 6454(92.25) | 10502(92.76) | 54.01 | 0.2889 | 9.3% | Fixed | 1.00 (0.99 to 1.02) | 0.8452 |
| **C Allele** | 2414 (46.70) | 3800 (45.90) | 51.43 | 0.3787 | 4.7% | Fixed | 1.00 (0.96 to 1.04) | 0.8450 |
| *CC Risk>1* | 1932 | 2888 |  |  |  |  |  |  |
| CC (12) | 233 (12.1) | 298 (10.3) | 4.43 | 0.9555 | 0% | Fixed | 1.28 (1.09 to 1.51) | 0.0025 |
| AC (12) | 894 (46.3) | 1284 (44.5) | 15.9 | 0.145 | 30.8% | Fixed | 1.05 (0.98 to 1.12) | 0.1463 |
| AA (12) | 805 (41.7) | 1306 (45.2) | 18.7 | 0.0664 | 41.2% | Fixed | 0.89 (0.84 to 0.95) | 0.0009 |
| CC+AC (12) | 1127 (58.3) | 1582 (54.8) | 18.2 | 0.0779 | 39.4% | Fixed | 1.09 (1.04 to 1.05) | 0.0007 |
| AA+AC (12) | 1669 (87.9) | 2590 (89.7) | 5.81 | 0.8859 | 0% | Fixed | 0.97 (0.95 to 0.99) | 0.0034 |
| *CC Risk<1* | 2622 | 3034 |  |  |  |  |  |  |
| CC (9) | 194 (7.4) | 286 (9.4) | 10.96 | 0.2042 | 27% | Fixed | 0.77 (0.65 to 0.92) | 0.0045 |
| AC (9) | 1050 (40.1) | 1313 (43.3) | 14.40 | 0.1088 | 37.5% | Fixed | 0.92 (0.86 to 0.98) | 0.0071 |
| AA (9) | 1378 (52.6) | 1435 (40.1) | 16.3 | 0.0377 | 51.1% | Random | 1.16 (1.07 to 1.26) | 0.0004 |
| CC+AC (9) | 1244 (47.4) | 1599 (52.7) | 14.7 | 0.0649 | 45.6% | Fixed | 0.89 (0.84 to 0.94) | <0.0001 |
| AA+AC (9) | 2428 (92.6) | 2748 (90.6) | 10.8 | 0.2121 | 26.1% | Fixed | 1.02 (1.01 to 1.04) | 0.0041 |
| *CC Risk~1* | 4314 | 8379 |  |  |  |  |  |  |
| CC (29) | 260 (6.0) | 452 (5.4) | 25.6 | 0.5944 | 0% | Fixed | 1.04 (0.90 to 1.21) | 0.5797 |
| AC (29) | 1510 (35.0) | 2930 (35.0) | 40.6 | 0.0584 | 31% | Fixed | 1.00 (0.95 to 1.05) | 0.8825 |
| AA (29) | 2544 (59.0) | 4997 (59.6) | 48.5 | 0.0095 | 42.2% | Random | 1.00 (0.96 to 1.05) | 0.9555 |
| CC+AC (29) | 1770 (41.0) | 3382 (40.4) | 43.4 | 0.0317 | 35.5 | Random | 1.01 (0.95 to 1.08) | 0.6525 |
| AA+AC (29) | 4054 (94.0) | 7927 (94.6) | 28.4 | 0.4449 | 1.3% | Fixed | 1.00 (0.99 to 1.01) | 0.5890 |
| *Note*:  *MTHFR =* methylenetetrahydrofolate reductase; CI = confidence interval; CC = *MTHFR* homozygous genotype CC; AC = *MTHFR* homozygous genotype AC; AA = MTHFR homozygous genotype AA; AC+CC = *MTHFR* homozygous genotype AC plus CC; AA+AC = *MTHFR* homozygous genotype AA plus AC.  Europe: Greece, Germany, Italy, Netherlands, United Kingdom, Portugal; America: Canada, Brazil; Middle East: Iran, Jordan, Turkey; East Asia: China, South Korea and Taiwan; South Asia: Singapore, Philippines, Indonesia and India; Africa: Egypt.  *CC Risk>1 countries included: Greece, India, Italy, Jordan, Netherlands, Philippines, Portugal and Singapore.*  *CC Risk<1countries included: Canada, Egypt, Indonesia, Taiwan and United Kingdom.*  *CC Risk~1 countries included: Brazil, China, Germany, Iran, South Korea and Turkey.* | | | | | | | | |
